# Three-Phase Heterojunction NiMo-Based Nano-Needle for Water Splitting at Industrial Alkaline Condition

**DOI:** 10.1007/s40820-021-00744-x

**Published:** 2021-12-09

**Authors:** Guangfu Qian, Jinli Chen, Tianqi Yu, Jiacheng Liu, Lin Luo, Shibin Yin

**Affiliations:** College of Chemistry and Chemical Engineering, State Key Laboratory of Processing for Non-Ferrous Metal and Featured Materials, Guangxi University, 100 Daxue Road, Nanning, 530004 People’s Republic of China

**Keywords:** Three-phase heterojunction, Interfacial electronic redistribution, Catalyst, Large current density, Water splitting

## Abstract

Constructing heterojunction is an effective strategy to develop high-performance non-precious-metal-based catalysts for electrochemical water splitting (WS). Herein, we design and prepare an N-doped-carbon-encapsulated Ni/MoO_2_ nano-needle with three-phase heterojunction (Ni/MoO_2_@CN) for accelerating the WS under industrial alkaline condition. Density functional theory calculations reveal that the electrons are redistributed at the three-phase heterojunction interface, which optimizes the adsorption energy of H- and O-containing intermediates to obtain the best Δ*G*_H*_ for hydrogen evolution reaction (HER) and decrease the Δ*G* value of rate-determining step for oxygen evolution reaction (OER), thus enhancing the HER/OER catalytic activity. Electrochemical results confirm that Ni/MoO_2_@CN exhibits good activity for HER (*ƞ*_*-*10_ = 33 mV, *ƞ*_*-*1000_ = 267 mV) and OER (*ƞ*_10_ = 250 mV, *ƞ*_1000_ = 420 mV). It shows a low potential of 1.86 V at 1000 mA cm^−2^ for WS in 6.0 M KOH solution at 60 °C and can steadily operate for 330 h. This good HER/OER performance can be attributed to the three-phase heterojunction with high intrinsic activity and the self-supporting nano-needle with more active sites, faster mass diffusion, and bubbles release. This work provides a unique idea for designing high efficiency catalytic materials for WS.

**Supplementary Information:**

The online version contains supplementary material available at 10.1007/s40820-021-00744-x.

## Highlights

- Three-phase heterojunction can adjust the ∆*G* of H/O-intermediates to boost catalytic activity.
- At ± 1000 mA cm^−2^, Ni/MoO_2_@CN exhibits low hydrogen/oxygen evolution reaction overpotentials (267/420 mV).
- Ni/MoO_2_@CN used as bifunctional electrodes can work at 1000 mA cm^−2^ for 330 h in 6.0 M KOH + 60 °C condition.

**Supplementary Information:**

The online version contains supplementary material available at 10.1007/s40820-021-00744-x.

## Graphical abstract

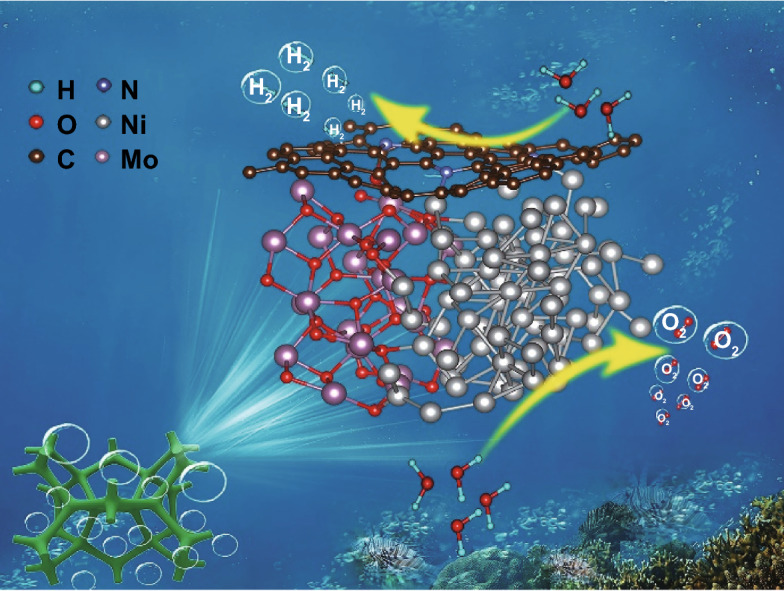

## Introduction

Electrochemical water splitting (WS) is considered as a green and convenient method for producing hydrogen (H_2_) [1–4]. However, the practical water electrolysis is limited by the sluggish kinetics at cathode (hydrogen evolution reaction, HER) and anode (oxygen evolution reaction, OER) [5–11]. For industrial WS catalyst, these requirements must be met: (1) stable and high active sites at large current density [12]; (2) continuous intensive gas evolution [13, 14]; (3) fast electron transfer [15, 16]; (4) low-cost and accessibility [17, 18]. Although some progress has been made, it is still a challenge to explore high-performance WS catalysts, especially in industrial environment.

The strategies, such as constructing heterojunction [19], fabricating carbon-encapsulating structure [20, 21], and manufacturing defects [22], are demonstrated to improve the WS performance of 3D transition-metals-based (TMB) catalysts. Notably, heterojunction is widely studied for enhancing the WS intrinsic activity through adjusting the electronic structure of catalysts [23, 24]. Mu et al*.* synthesized Mo-doped Ni_3_S_2_/Ni_*x*_P_*y*_ heterojunction, which optimizes the absorption energy of H- and O-containing intermediates to promote the WS catalytic process [25]. Jiao et al. [26] prepared the heterojunction of Co and nitrogen-rich nitride, which induces its interfacial electronic redistribution to obtain the optimal H* absorption and decrease the dissociation energy barrier.

Furthermore, carbon-encapsulated 3D TMB materials as a kind of heterojunction can not only modulate the electronic structure to boost HER/OER catalytic activity, but also avoid metal corrosion in harsh electrolyte to improve the stability [27]. Deng et al. [28] proved that the surface electronic structure of FeNi alloy with ultrathin graphene layer is adjusted to obtain the good OER performance. Despite the significant progress, most of them are still far from the practical current density (≥ 500 mA cm^−2^). In our previous work, the graphene-coated NiCo alloy coupled with NiCoMoO and self-supported on 3D nickel foam (NiCo@C–NiCoMoO/NF) was synthesized to improve the intrinsic activity and stability at large current density (≥ 500 mA cm^−2^), which is different from other carbon-encapsulated 3D TMB materials due to the unique metal oxide anchoring alloy structure. Nevertheless, the understanding of its active sites is still not deep enough. Thus, developing 3D TMB materials with excellent WS performance at large current density and investigating the mechanism of its high intrinsic activity are significant but challenging.

Besides, in order to improve WS stability at large current density, constructing a reasonable structure (nanowires, nanosheets, etc*.*) on 3D porous substrate (e.g., NF) is significant, which can accelerate the mass transfer and bubbles escape during the electrochemical reaction process, and provide large specific surface area to expose abundant active sites [15, 29]. MoS_2_/Ni_3_S_2_ heterostructure nanowires self-supported on NF were constructed by Cheng et al*.* [30]. It exhibits a small overpotential of 182 mV at 500 mA cm^−2^ for HER, which is comparable to Pt/C.

Inspired by the above strategies, the N-doped-carbon-coated Ni/MoO_2_ nano-needle with three-phase heterojunction (Ni/MoO_2_@CN) self-supported on 3D porous NF is rationally engineered and synthesized for the first time, and the HER/OER intrinsic activity is further investigated by density functional theory (DFT) calculations. This novel structure has the following advantages: (1) The electronic structure of N-doped-carbon (CN), Ni, and MoO_2_ can be optimized at three-phase heterojunction interface for adjusting the adsorption energy of H- and O-containing intermediates, which can be beneficial to obtain the best H* Gibbs free energy (Δ*G*_H*_) for HER and decrease the Gibbs free energy (Δ*G*) value of rate-determining step (RDS) for OER, thus enhancing the HER/OER intrinsic activity and electron transfer ability under a large current density [31]. (2) CN can prevent metal corrosion in electrolyte to boost catalytic stability [27, 32]. (3) Nano-needles self-supported on 3D NF with meso/macroporous structure can provide large specific surface area to expose more active sites for reducing the energy barriers of WS [33]. Furthermore, it is beneficial to mass diffusion and bubbles release at large current density, thus improving the WS performance [34].

Benefitting from the synergistic effect among the CN, Ni, and MoO_2_ at the three-phase heterojunction interface, as well as the unique self-supporting nano-needle structure, the prepared Ni/MoO_2_@CN exhibits low overpotentials at ± 10 and  ± ﻿1000﻿ mA cm^−2^ for HER (*ƞ*_*-*10_ = 33 mV, *ƞ*_−1000_ = 267 mV) and OER (*ƞ*_10_ = 250 mV, *ƞ*_1000_ = 420 mV). As for WS, it shows a low potential (1.86 V) at 1000 mA cm^−2^ in 6.0 M KOH solution at 60 °C with an ignorable depletion of activity after 330 h testing, showing excellent stability. Therefore, this work provides a promising catalyst for WS, and it sheds light on developing highly efficient non-precious-metal materials for industrial water electrolysis.

## Result and Discussion

### DFT Calculations for HER and OER

**Fig. 1:**
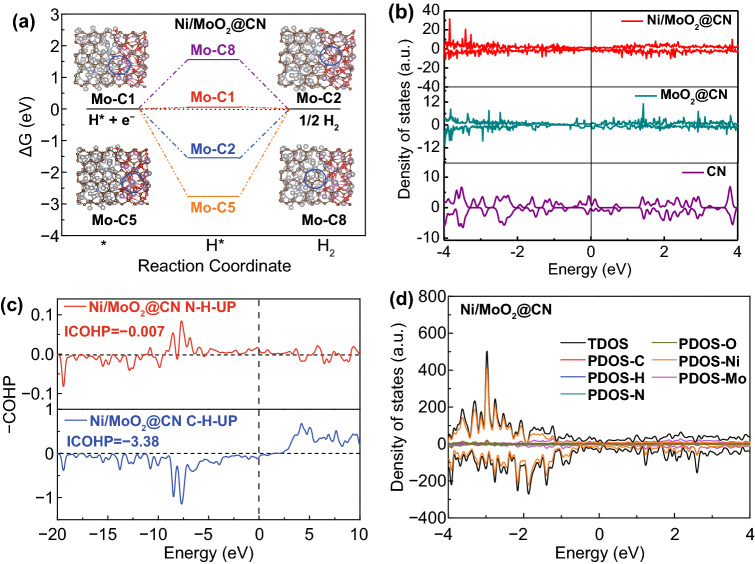
**a** Δ*G*_H*_ calculated at different adsorb sites for Ni/MoO_2_@CN model, insert: schematic illustration of H adsorption; **b** PDOS analysis of C for all samples; **c** COHP; and **d** PDOS analysis for the Ni/MoO_2_@CN model with the H atom adsorbed on the sites

Firstly, the computational models of Ni/MoO_2_@CN, Ni/MoO_2_, Ni@CN, MoO_2_@CN, and CN are designed (the details are displayed in Fig. S1). Then, the three-phase heterojunction interface synergy effect and HER activity are studied via DFT computations. Particularly, the Δ*G*_H*_ and electron distribution for the active atoms are investigated by DFT and density of states (DOS) [35]. Hence, we consider all possible active sites of Ni/MoO_2_@CN for HER and calculate their Δ*G*_H*_ (Figs. 1a and S2). As shown in Fig. 1a, the Δ*G*_H*_ of Ni/MoO_2_@CN (0.056 eV) on the Mo-ortho-C between Ni and MoO_2_ interface (named as Mo-C1) is close to zero, which is superior to other active sites in Ni/MoO_2_@CN model (Figs. 1a and S3). Furthermore, the Δ*G*_H*_ value of Mo-C1 in Ni/MoO_2_@CN model is better than the ortho-C of MoO_2_@CN, Ni@CN, and CN models (Figs. S4–S9), indicating that the three-phase heterojunction interface can effectively adjust the H adsorption energy for ortho-C to obtain the highest HER intrinsic activity.

Afterward, the DOS of CN, MoO_2_@CN, and Ni/MoO_2_@CN models are used to further confirm the electronic structure of C at the three-phase heterojunction interface. As displayed in Fig. 1b, the strength of this ordinate for CN, MoO_2_@CN, and Ni/MoO_2_@CN models indicates that the introduction of Ni species can construct the three-phase heterojunction interface to adjust the electronic structure of CN, which optimizes the Δ*G*_H*_ of HER. Besides, for revealing the bond capabilities, the crystal orbital Hamilton population (COHP) derived from partial DOS (PDOS) calculation (Fig. 1c, d) are implemented [36]. The energy integral of COHP (ICOHP) of C–H for Ni/MoO_2_@CN is − 3.38 (Fig. 1c), which is larger than those in other models (Figs. S5, S7, and S9), suggesting that it has stronger hydrogen adsorption to obtain higher HER catalytic activity [37].

**Fig. 2:**
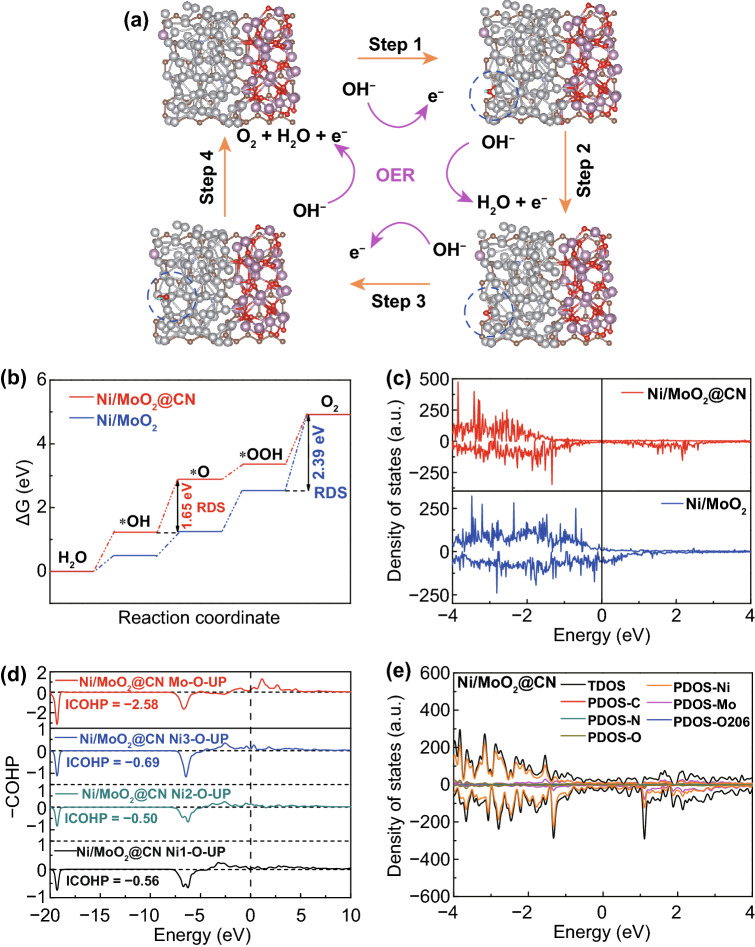
**a** Schematic illustration of the *OH, *O, and *OOH adsorption for Ni/MoO_2_@CN model; **b** OER reaction pathway for Ni/MoO_2_@CN model; **c** PDOS analysis of Ni for all samples; **d** COHP; and **e** PDOS analysis for the Ni/MoO_2_@CN model with the O atom adsorbed on the sites

Subsequently, Fig. 2a shows the four-step mechanism processes for OER in alkaline media [38]. The surface of Ni/MoO_2_@CN (Fig. 2a), Ni/MoO_2_ (Fig. S10), Ni@CN (Fig. S11), MoO_2_@CN (Fig. S12), and CN (Fig. S13) models displays the adsorption of OER intermediates. During the four OER reaction processes, the Δ*G* is employed to investigate the intrinsic activity, and the largest Δ*G* value indicates the rate-determining step (RDS) [39]. As shown in Fig. 2b, step 4 is RDS for Ni/MoO_2_, which exhibits Δ*G* value of 2.39 eV. For Ni/MoO_2_@CN, we combine CN with Ni/MoO_2_ to form the three-phase heterogeneous junction interface, the RDS is changed from step 4 to step 2, and its Δ*G* value is reduced to 1.65 eV. The smaller Δ*G* value of Ni/MoO_2_@CN implies the faster reaction kinetics to obtain lower potential during the OER process, and it is better than other catalysts in this work (Figs. S14–S16). Besides, it is found that the Ni *d*-band center of Ni/MoO_2_@CN exhibits a negative shift compared with Ni/MoO_2_ (Fig. 2c), which is also good for adjusting the adsorption of O-intermediates. Meanwhile, the PDOS results (Fig. 2c) demonstrate that the electrons are transferred from Ni to CN, indicating that the three-phase heterojunction formed by CN and Ni/MoO_2_ exists electron interaction, which could enhance the intrinsic activity for OER.

The COHP (Fig. 2d) is used to uncover the bond capabilities, which is originated from PDOS calculation (Fig. 2e) [40]. When CN combines with Ni/MoO_2_ to form the three-phase heterojunction, the strength of interaction between Ni and O can be changed. As shown in Figs. 2d  and S17a, the ICOHP (− 0.69, − 0.50, − 0.56) values of Ni/MoO_2_@CN for Ni suggest that it possesses better interaction between Ni and O, in relative to ICOHP values (− 0.54, − 0.54, − 0.54) of Ni/MoO_2_. Furthermore, the Mo atom has good oxygen adsorption capacity (− 2.58) at the three-phase heterojunction interface to provide more O-intermediates to boost the OER intrinsic activity. Thus, the COHP results indicate that the three-phase heterojunction interface composed of Ni, MoO_2_, and CN can effectively optimize the desorption of O-containing intermediates to enhance the OER intrinsic activity.

### Physicochemical Characterization

**Fig. 3:**
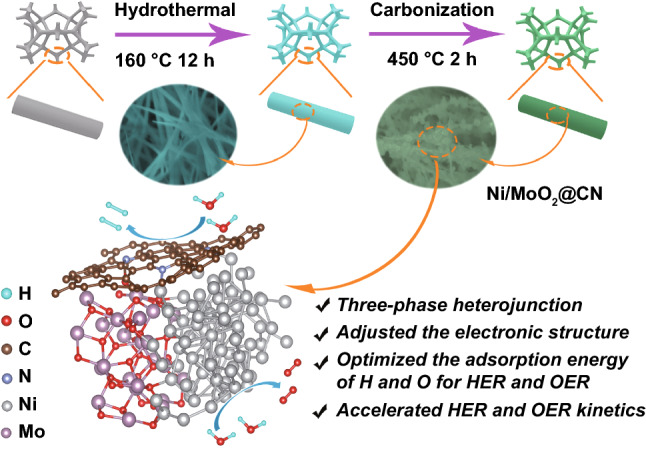
Synthesis diagram of Ni/MoO_2_@CN

Guided by the DFT predictions, the facile two-step methods (Fig. 3, the detailed steps are shown in Supplementary Information) are used to synthesize the CN layer coated Ni/MoO_2_ nano-needle with three-phase heterojunction (Ni/MoO_2_@CN) self-supported on 3D porous NF. The powder used for X-ray diffraction (XRD) characterization is obtained from NF that has been treated by ultrasonication rather than directly using NF samples. As shown in Supplementary Figure S18, firstly, the NiMoO_4_ nano-needle is grown on NF via the solvothermal method, which can be determined by XRD (Supplementary Figure S19). Then, the NiMoO_4_ nano-needle is reduced by the high temperature (450 °C) calcination in a mixed atmosphere (5% H_2_ + 95% Ar) to form the Ni nanoparticle and MoO_2_ nano-needle, and this is confirmed by XRD and scanning electron microscopy (SEM, Fig. S20–S21). The reason can be attributed to the different enthalpy of Ni and Mo [41]. Besides, Raman proves the existing of CN, which can be ascribed to the reduction of organic carbon to CN [the ratio of area D and G (Area_D_ and Area_G_) is 1.20], this is because the catalysis of Ni atoms (Fig. S22c) [22, 42]. Thus, these results suggest that the catalyst is composed of CN, Ni, and MoO_2_.

Subsequently, the XRD, Raman, and SEM images obtained by annealing the NiMoO_4_ nano-needle at 350 and 550 °C are shown in Figs. S22–S23, respectively. When it calcined at 350 °C, the characteristic peak intensities of Ni and MoO_2_ are too weak. In this case, the morphology of nano-needle is smooth, and the ratio of Area_D_/Area_G_ is 1.10 with less defects of CN. When it calcined at 550 °C, the characteristic peaks of Ni and MoO_2_ are replaced by the MoNi alloy, and the morphology of nano-needle is destroyed, under the circumstances, the ratio of Area_D_/Area_G_ is 1.15. Therefore, these results indicate that the temperature plays an important role on the formation of this catalyst.

Then, summarizing the results of XRD, Raman, and SEM (Figs. S22–S23) obtained at different temperatures, the following reason for the formation of catalyst can be proposed: The different enthalpy of Ni and Mo can lead to the segregation of Ni atoms from NiMoO_4_ to form Ni as well as the reduction of MoO_4_^2−^ to MoO_2_ at 450 °C, and the segregated Ni can catalyze the organic carbon to form CN. However, when the NiMoO_4_ nano-needle annealed at 350 °C, due to the low temperature, there is no enough energy to reduce NiMoO_4_ forming Ni and MoO_2_, and the surface organic carbon cannot form CN. And when it calcined at 550 °C, due to the high temperature, the Ni and Mo atoms segregate from the NiMoO_4_ to form the NiMo alloy, but organic carbon cannot form the CN on its surface, mainly attributed to the rapid reduction at such high temperature. Therefore, the NiMoO_4_ calcined at 350 and 550 °C exhibits lower HER/OER intrinsic activity (Figs. S29 and S41).

**Fig. 4:**
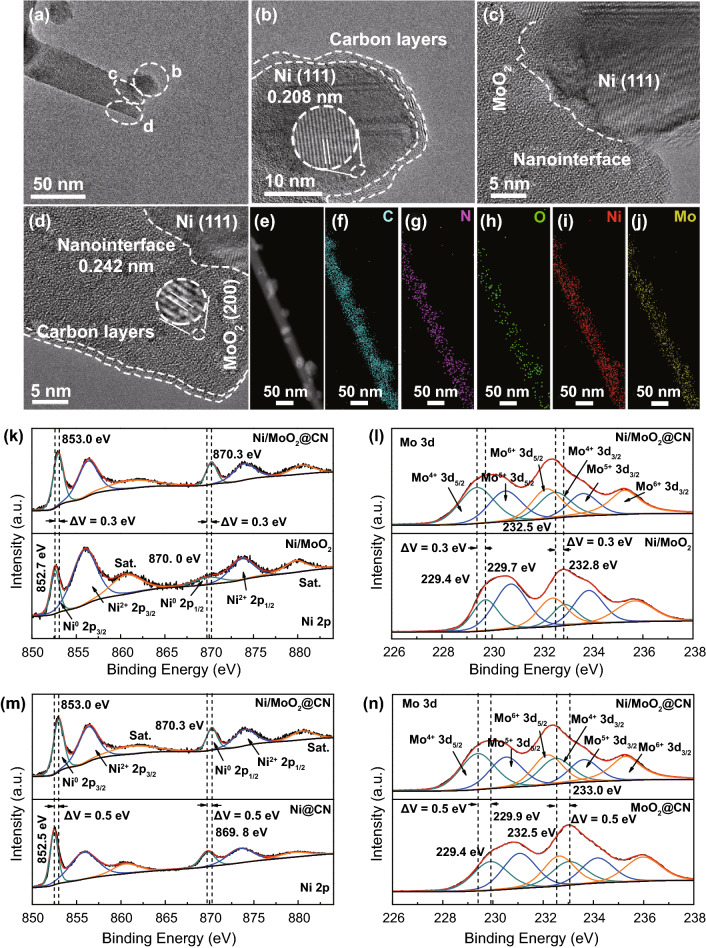
**a**–**j** TEM, HRTEM, HAADF-STEM, and elemental mapping images of Ni/MoO_2_@CN; **k**–**n** HRXPS of Ni 2*p* and Mo 3*d* spectra for Ni/MoO_2_@CN, Ni/MoO_2_, Ni@CN, and MoO_2_@CN

Subsequently, the transmission electron microscopy (TEM) and high-resolution TEM (HRTEM) are employed to reveal the three-phase heterojunction and lattice fringe of Ni/MoO_2_@CN. As shown in Fig. 4a–c, the 0.208 nm (111) of Ni nanoparticle couples with the 0.242 nm (200) of MoO_2_ nano-needle (consistent with XRD results). Obviously, the carbon layers are coated on the surface of Ni (≈ 3 layers, Fig. 4b) and MoO_2_ (≈ 2 layers, Fig. 4d), which can efficiently optimize the electron distribution to enhance the HER/OER intrinsic activity, and this is also confirmed by DFT [43, 44]. Furthermore, carbon layers can avoid metal corrosion in electrolyte to improve the electrochemical stability [27]. More importantly, a clear heterojunction exists between the Ni nanoparticle and MoO_2_ nano-needle (Fig. 4c, d), which can realize the redistribution of electrons at the interface for further optimizing the adsorption energy of H- and O-containing intermediates to boost the HER/OER intrinsic activity [25]. Additionally, heterojunction can enhance the electron transfer ability to improve the WS performance at large current density [45]. The high-angle annular dark-field scanning TEM (HAADF-STEM) and elemental mapping images sugggest the uniform distribution of C, N, O, Ni, and Mo on the Ni/MoO_2_@CN (Fig. 4e–j).

X-ray photoelectron spectroscopy (XPS) is used to evaluate the elemental compositions and valance states. XPS survey spectrum of Ni/MoO_2_@CN is shown in Fig. S24a, which confirms the existence of Ni, C, Mo, N, and O elements. In Fig. S24b, c, HRXPS of O 1*s* and C 1*s* is located at Mo–O (530.8 eV), absorbed-O (532.1 eV), C=O (288.8 eV), C–N (286.3 eV), and C–C (284.8 eV). For N 1*s* spectrum, the peaks at 396.1 and 398.6 eV belong to Mo 3*p* and pyridinic-N (Fig. S24d), as known, the doping of N atoms into carbon layer can enhance the HER/OER intrinsic activity [46, 47]. The Ni 2*p* and Mo 3*d* of Ni/MoO_2_@CN show a positive shift (≈ 0.3 eV, Fig. 4k and Table S1) and negative shift (≈ 0.3 eV, Fig. 4l and Table S2) relative to Ni/MoO_2_, respectively, displaying that the CN affects the electronic structure of Ni and MoO_2_, and this is in agreement with the DFT predictions.

Furthermore, Ni 2*p* spectrum of Ni/MoO_2_@CN presents a positive shift (≈ 0.5 eV, Fig. 4m and Table S1) relative to Ni@CN, showing that the introduction of MoO_2_ can adjust the electronic structure of Ni@CN. In Fig. 4n and Table S2, Mo 3*d* spectrum of Ni/MoO_2_@CN exhibits a negative shift (≈ 0.5 eV) as against to MoO_2_@CN, which suggests that the electronic structure of Mo 3*d* for MoO_2_@CN is also affected, because it is coupled with Ni@CN to form the three-phase heterojunction interface. In contrast, as displayed in Fig. S25a, b, Ni 2*p*, and Mo 3*d* peaks of Ni@CN + MoO_2_@CN hybrids (mixing the Ni@CN and MoO_2_@CN together) are the same as Ni@CN and MoO_2_@CN, respectively, indicating that the mechanical mixing cannot affect the binding energy. However, compared with the Ni@CN + MoO_2_@CN hybrids, the Ni 2*p* and Mo 3*d* peaks of Ni/MoO_2_@CN present positive and negative shift, respectively. These results demonstrate that coupling Ni@CN with MoO_2_@CN to form the three-phase heterojunction interface can lead to the strong electronic interaction, thus adjusting the absorption/desorption of H/O-intermediates to enhance the intrinsic activity [48], which is consistent with DFT calculation results.

It can be seen from the above results that there is indeed a three-phase heterojunction between Ni, MoO_2_, and CN. This unique three-phase heterojunction can make the interfacial electronic redistribution of Ni, MoO_2_, and CN, thus providing lots of defects to form abundant active sites for reducing the WS reaction energy barrier. Furthermore, the nano-needle self-supporting on 3D NF can expose more active sites to further increase WS catalytic activity, and it is also beneficial for mass diffusion at large current density to improve the WS performance.

### HER Catalytic Performance

**Fig. 5:**
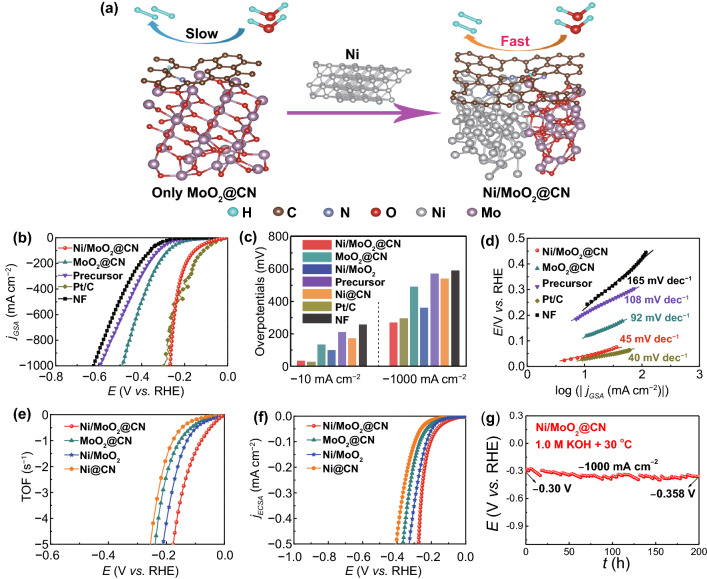
**a** Schematic diagram of HER mechanism: Ni/MoO_2_@CN exhibits better HER kinetics compared to MoO_2_@CN; **b** LSV curves; **c** comparison of overpotentials; **d** Tafel plots; **e** TOF curves; **f** ECSA-normalized LSV curves of as-prepared samples; **g** CP curve of Ni/MoO_2_@CN

Based on the guidance of DFT results and the unique three-phase heterojunction of Ni/MoO_2_@CN, HER mechanism can be illustrated in Fig. 5a, and the HER activity is evaluated by the linear sweep voltammetry (LSV) curves with/without *iR*-correction (Fig. S26). As displayed in Fig. 5b, c, the Ni/MoO_2_@CN possesses good HER activity with low overpotentials (*η*_*−*10_ = 33 mV, *η*_*−*1000_ = 267 mV), which is better than that of MoO_2_@CN (*η*_*−*10_ = 134 mV, *η*_*−*1000_ = 430 mV). This result suggests that the introduction of Ni into MoO_2_@CN to form the three-phase heterojunction of Ni/MoO_2_@CN can greatly enhance the HER activity, and it is consistent with the DFT results. It is also better than that of precursor (*η*_*−*10_ = 210 mV, *η*_*−*1000_ = 570 mV), NF (*η*_*−*10_ = 256 mV, *η*_*−*1000_ = 590 mV), and close to Pt/C (*η*_*−*10_ = 25 mV, *η*_*−*1000_ = 296 mV), and superior to most of the recently reported catalysts (Fig. S27 and Table S3). Furthermore, Ni/MoO_2_@CN shows smaller overpotentials compared with Ni@CN (*η*_*−*10_ = 173 mV, *η*_*−*1000_ = 474 mV) in Figs. 5c and S28a. This proves that the three-phase heterojunction of Ni/MoO_2_@CN synthesized by combining MoO_2_ with Ni@CN leads to further optimize the electronic structure of CN for obtaining an outstanding HER intrinsic activity, and it agrees with DFT results. Meanwhile, Ni/MoO_2_ needs higher overpotentials (*η*_*−*10_ = 100 mV, *η*_*−*1000_ = 358 mV) compared with Ni/MoO_2_@CN in Figs. 5c and S28a, implying that the CN decorated Ni/MoO_2_ can reduce the HER reaction barrier by optimizing the Δ*G*_H*_.

Through above results comparing of Ni/MoO_2_@CN, Ni/MoO_2_, Ni@CN, and Ni/MoO_2_, we can conclude that the interfacial charge redistribution at three-phase heterojunction is good for HER, and the Ni, MoO_2_, and CN are all indispensable. Moreover, the sample with a Ni/Mo molar ratio of 1:7 (Fig. S29) and the NiMoO_4_ annealed at 450 °C (Supplementary Figure S30) exhibits higher HER intrinsic activity, which indicates that the Ni/Mo molar ratio as well as the calcination temperature has an important impact on the HER intrinsic activity.

Figures 5d and S28b show Tafel plots originated from the LSV curves. The Ni/MoO_2_@CN exhibits a lower Tafel slope of 45 mV dec^−1^ than those of MoO_2_@CN (92 mV dec^−1^), Ni@CN (85 mV dec^−1^), Ni/MoO_2_ (81 mV dec^−1^), precursor (108 mV dec^−1^), NF (165 mV dec^−1^), and like Pt/C (40 mV dec^−1^), indicating that Ni/MoO_2_@CN obtains faster HER catalytic kinetics. Then, the charge transfer kinetics is analyzed by electrochemical impedance spectroscopy (Fig. S31), and Ni/MoO_2_@CN has the smallest charge transfer resistance (*R*_ct_) among all samples, implying the fastest charge transfer rate of HER. The superiority of HER reaction kinetics and charge transfer can be ascribed to interface electronic transmission at the three-phase heterojunction among CN, Ni, and MoO_2_.

Besides, the turnover frequency (TOF) and electrochemical surface area (ECSA) are used to explore the HER intrinsic activity and the number of active sites, respectively [49]. Cyclic voltammetry (CV) method is used to study the TOF of HER (Fig. S32) [50]. In Fig. 5e and Table S4, Ni/MoO_2_@CN possesses the largest TOF value (1.45 s^−1^) at the overpotential of 100 mV, as compared with MoO_2_@CN (0.38 s^−1^), Ni@CN (0.19 s^−1^), and Ni/MoO_2_ (0.53 s^−1^), suggesting that Ni/MoO_2_@CN has the highest catalytic activity. Besides, it also has the highest TOF values at other overpotentials (Table S5), and it is better than most of the previously reported results in literature (Fig. S33 and Table S6).

ECSA can be evaluated by electrochemical double-layer capacitances (*C*_dl_), due to the positive correlation between ECSA and *C*_dl_ (Fig. S34a–d) [42]. Obviously, Ni/MoO_2_@CN exhibits the largest *C*_dl_ (97.94 mF) in Fig. S34e, which implies that it has the highest density of active sites to accelerate the HER process, and it is also reflected in the ECSA-normalized LSV curves (Fig. 5f). The highest intrinsic activity can be due to the interfacial charge redistribution at the three-phase heterojunction interface, which obtains suitable adsorption energy of H and O-containing intermediates. Besides, the self-supporting nano-needle can provide a large specific surface area to expose more active sites.

Stability is another vital index to evaluate catalyst performance [51], especially at large current density. Ni/MoO_2_@CN displays outstanding durability at − 1000 mA cm^−2^ for 200 h with stable release of H_2_ bubbles, which is tested by the chronopotentiometry (CP) method (Fig. 5g). Furthermore, the activity before and after the CP test has negligible changes (Fig. S35), implying its good stability. Subsequently, the SEM images (Fig. S36) and HRXPS spectra (Fig. S37) of Ni/MoO_2_@CN after the CP test are also obtained, and they almost maintain the original state. The better stability can be attributed to the following reasons: (1) The interfacial charge redistribution at three-phase heterojunction for Ni/MoO_2_@CN can accelerate the electron transfer at large current density; (2) Carbon layers could avoid the dissolution of metal in harsh solution; (3) Self-supporting nano-needle enhances the wettability of catalyst to effectively boost the mass diffusion and bubbles release.

### OER Catalytic Performance

**Fig. 6:**
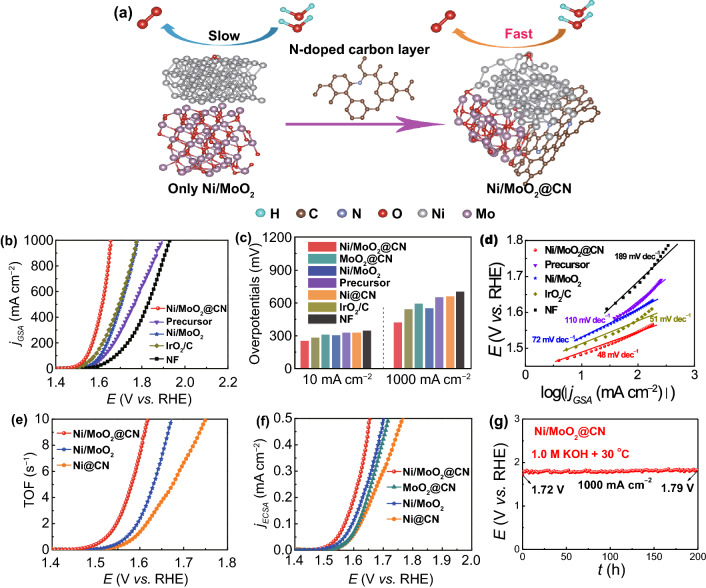
**a** Schematic diagram of OER mechanism: Ni/MoO_2_@CN exhibits better OER kinetics compared to Ni/MoO_2_; **b** LSV curves; **c** comparison of overpotentials; **d** Tafel plots; **e** TOF curves; **f** ECSA-normalized LSV curves of as-prepared samples; **g** CP curve of Ni/MoO_2_@CN

The Ni/NiMoO_2_@CN not only has outstanding HER performance, but also exhibits good OER performance under the same condition; the schematic diagram of the OER mechanism is displayed in Fig. 6a. The OER LSV curves of Ni/MoO_2_@CN with/without *iR-*correction are presented in Fig. S38. As shown in Fig. 6b, c, Ni/MoO_2_@CN shows higher activity (*η*_10_ = 250 mV, *η*_1000_ = 420 mV) than Ni/MoO_2_ (*η*_10_ = 300 mV, *η*_1000_ = 550 mV), which indicates that the decoration of CN to Ni/NiMoO_2_ can effectively enhance the OER intrinsic activity. Meanwhile, the overpotentials of Ni/MoO_2_@CN are also smaller than that of MoO_2_@CN (*η*_10_ = 310 mV, *η*_1000_ = 590 mV) and Ni@CN (*η*_10_ = 323 mV, *η*_1000_ = 660 mV) in Figs. 6c and S39a. These results indicate that the electronic structures of Ni, Mo, and CN are adjusted by interfacial electronic redistribution at the three-phase heterojunction to further optimize the adsorption of O-intermediates; this is also consistent with the DFT results. Besides, as shown in Fig. 6b, c, the Ni/MoO_2_@CN exhibits smaller overpotentials than NF (*η*_10_ = 345 mV, *η*_1000_ = 700 mV), precursor (*η*_10_ = 327 mV, *η*_1000_ = 650 mV), IrO_2_/C (*η*_10_ = 280 mV, *η*_1000_ = 540 mV), and even superior to most of the recently reported catalysts (Fig. S40 and Table S7). Moreover, the sample with a Ni/Mo molar ratio of 1:7 (Fig. S41) and the NiMoO_4_ annealed at 450 °C (Fig. S42) displays the lowest OER overpotential, suggesting that the Ni/Mo molar ratio and calcination temperature play an important role on the OER intrinsic activity.

In addition, the Tafel slope and EIS are used to assess the kinetics of OER (Figs. 6d, S39b and S43). Obviously, Ni/MoO_2_@CN displays the smallest Tafel slope and smallest *R*_ct_ value among all catalysts, which implies its high electron transfer rate and fast kinetics for OER at the three-phase heterojunction interface. Subsequently, the good OER intrinsic activity of Ni/MoO_2_@CN is also verified by TOF. The active surface redox sites method is used to study the TOF for OER by calculating the redox surface sites of Ni^2+^/Ni^3+^ without the capacitive current (Fig. S44) [50]. In Fig. 6e and Table S8, the TOF value of Ni/MoO_2_@CN (1.23 s^−1^) is higher than Ni/MoO_2_ (0.28 s^−1^) and Ni@CN (0.14 s^−1^) at the overpotential of 300 mV, suggesting its higher intrinsic activity. Besides, it also has higher TOF values at other overpotentials (Table S9), which is better than most of the reported works (Fig. S45 and Table S10). The current density is normalized by ECSA to further reflect the highest intrinsic activity of Ni/MoO_2_@CN (Fig. 6f). The remarkable OER catalytic activity can be ascribed to the following reasons: (1) Interfacial electronic redistribution at three-phase heterojunction interface can be beneficial to optimize the adsorption energy of O-containing intermediates; (2) Interfacial electronic redistribution also can accelerate the charge transfer at large current density to enhance OER activity; (3) Self-supporting nano-needle has a large specific surface area to provide more active sites for boosting the OER performance.

Subsequently, its stability is studied by CP method. Impressively, the Ni/MoO_2_@CN maintains good stability for 200 h at 1000 mA cm^−2^ (Fig. 6g); the change of potential is about 70 mV. To investigate its outstanding durability, LSV curves and Nyquist plots before and after stability test are displayed in Fig. S46; the potential at 1000 mA cm^−2^ and *R*_ct_ value at 1.5 V have minor changes. The reason for the good OER stability should be the same as HER. Then, the change of morphology and elements states after the CP test are studied by SEM and HRXPS. In Fig. S47, SEM images of Ni/MoO_2_@CN can keep the primary appearance, indicating its good stability. The HRXPS spectrum of Ni^0^ disappeared (Fig. S48), implying that the Ni^0^ is oxidized to NiOOH [52], and the Mo^4+^ and Mo^5+^ are also oxidized to Mo^6+^, which is a common phenomenon during OER process [42]. As reported in literatures [52–54], the generated NiOOH can combine with the Ni to form the heterojunction interface between the NiOOH and Ni during OER process, thus further enhancing the OER intrinsic activity. Meanwhile, according to literatures, the generated Mo^6+^ can be beneficial to the conversion of Ni^2+^ to Ni^3+^ and produce the NiOOH for enhancing the OER intrinsic activity [55, 56].

### WS Catalytic Performance

**Fig. 7:**
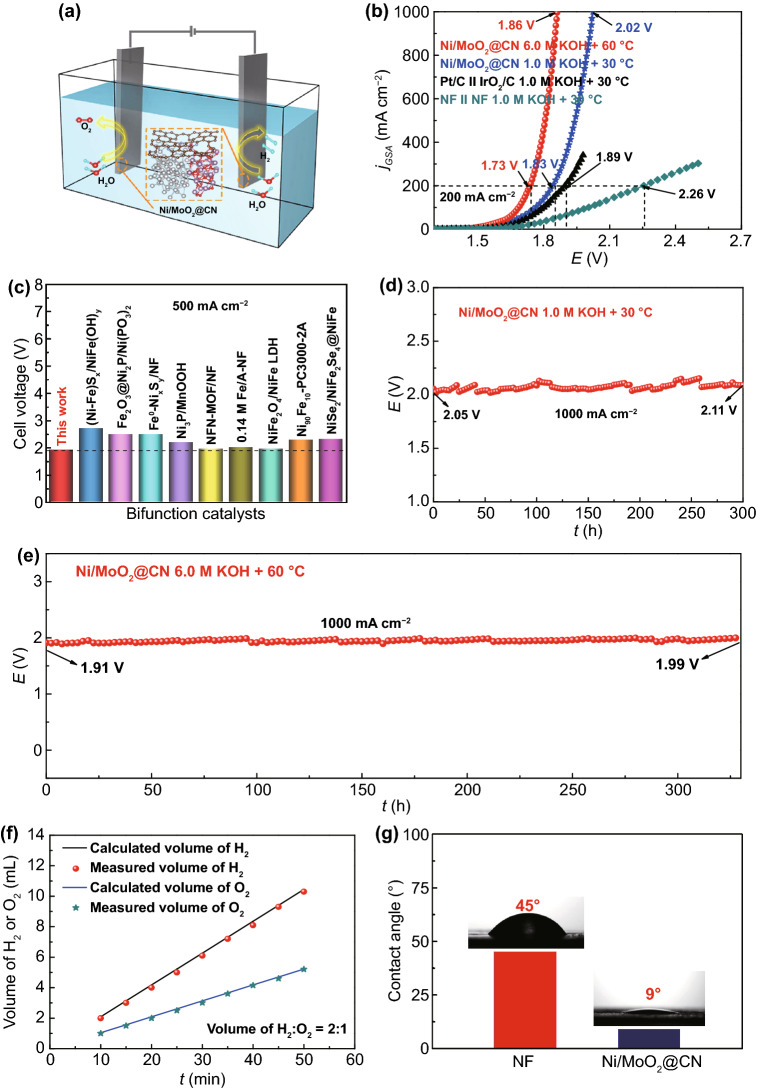
**a** Schematic diagram of WS for Ni/MoO_2_@CN; **b** LSV curves of the samples in 1.0 M KOH + 30 °C, and LSV curve of Ni/MoO_2_@CN in 6.0 M KOH + 60 °C; **c** comparison of potentials at 500 mA cm^−2^ of Ni/MoO_2_@CN and reported catalysts; CP curves of Ni/MoO_2_@CN **d** at 1.0 M KOH + 30 °C and **e** 6.0 M KOH + 60 °C; **f** Volume of H_2_ and O_2_ measured at 30.0 mA as a function of time for Ni/MoO_2_@CN; **g** CA of NF and Ni/MoO_2_@CN under 1.0 M KOH. Insert: optical photographs

In considering of the outstanding HER and OER performance of Ni/MoO_2_@CN, the WS performance of Ni/MoO_2_@CN is also evaluated by two-electrode system to simulate the actual application, and the schematic diagram is shown in Fig. 7a. For comparison, the WS intrinsic activity of Pt/C//IrO_2_/C and NF//NF are also measured by the same method with Ni/MoO_2_@CN. Under 1.0 M KOH + 30 °C condition, the LSV curve of Ni/MoO_2_@CN (1.83 V) as cathode and anode electrodes display a higher activity than Pt/C//IrO_2_/C (1.89 V) and NF//NF (2.26 V) at 200 mA cm^−2^ (Fig. 7b). Interestingly, the Ni/MoO_2_@CN as bifunctional electrodes can drive the large current density of 500 and 1000 mA cm^−2^ while only requiring 1.92 and 2.02 V, and it is better than most of the reported literatures at 500 mA cm^−2^ (Fig. 7c) [57–65]. Furthermore, it can maintain for 300 h at 1000 mA cm^−2^ (Fig. 7d), exhibiting its excellent stability.

In addition, in order to further evaluate the industrial application potential of Ni/MoO_2_@CN, it is applied to WS under the simulated industrial condition (6.0 M KOH + 60 °C). As shown in Fig. 7b, it only requires 1.86 V for Ni/MoO_2_@CN to reach 1000 mA cm^−2^ when used as cathode and anode electrodes. It can work stably for 330 h at 1000 mA cm^−2^ with negligible potential change (Fig. 7e), implying that it is a potential material for large-scale hydrogen production. The good WS performance of Ni/MoO_2_@CN under the large current density of industrial condition can be attributed to the three-phase heterojunction with high HER/OER intrinsic activity and the self-supporting nano-needle structure with more active sites, faster bubbles desorption, and the diffusion of electrolyte.

Subsequently, the Faradic efficiency and contact angles (CA) of Ni/MoO_2_@CN are surveyed (Figs. 7f, g and S49). The volume–time curve for H_2_ and O_2_ is obtained by water drainage method at 0, 10, 15, 20, 25, 30, 35, 40, 45, and 50 min measured at 30.0 mA. The volume–time curve for H_2_ and O_2_ is about 2:1, and they are close to the theoretical values, indicating the nearly 100% Faradic efficiency. Besides, the wettability of catalysts is very important for WS. The CA of Ni/MoO_2_@CN and NF with 1.0 M KOH solution are shown in Fig. 7g. Obviously, the CA of Ni/MoO_2_@CN (9°) is smaller than NF (45°), suggesting the good gas release and mass transfer. Then, as shown in Fig. S50, the size of H_2_ bubbles on NF electrode is much larger than it on Ni/MoO_2_@CN electrode, indicating that the H_2_ bubbles are easier to desorb from the surface of Ni/MoO_2_@CN. Thus, the above results suggest that Ni/MoO_2_@CN has good hydrophilicity to enhance the contact with electrolyte and facilitate bubbles desorption for improving the performance of WS.

## Conclusions

In short, the CN layers encapsulated Ni/MoO_2_ nano-needle with three-phase heterojunction is successfully engineered and synthesized. Interestingly, as predicted by DFT results, the electrons are redistributed among the CN, Ni, and MoO_2_ for optimizing the adsorption energy of H- and O-containing intermediates in order to obtain the best Δ*G*_H*_ for HER and decrease the Δ*G* value of RDS for OER, thus enhancing the HER/OER intrinsic activity. The physical and electrochemical results are in good agreement with the theoretical predictions. Compared with the XPS results of Ni/MoO_2_, Ni@CN, and MoO_2_@CN, the peaks of Ni 2*p* and Mo 3*d* for Ni/MoO_2_@CN show negative/positive shift, which further demonstrate that there is a strong interaction between CN, Ni, and MoO_2_. Therefore, the Ni/MoO_2_@CN exhibits good activity for HER (*ƞ*_*−*10_ = 33 mV) and OER (*ƞ*_10_ = 250 mV) compared with Ni/MoO_2_ (HER: *ƞ*_*−*10_ = 100 mV, OER: *ƞ*_10_ = 300 mV), MoO_2_@CN (HER: *ƞ*_*−*10_ = 134 mV, OER: *ƞ*_10_ = 310 mV), and Ni@CN (HER: *ƞ*_*−*10_ = 173 mV, OER: *ƞ*_10_ = 323 mV). Meanwhile, the three-phase heterojunction can facilitate electronic transfer at large current density and the carbon layers can avoid the metal dissolution in harsh solution. Self-supporting nano-needle can enhance the wettability of catalyst to effectively boost the mass diffusion and bubbles release. Therefore, it can steadily operate for 330 h at 1000 mA cm^−2^ for WS (6.0 M KOH + 60 °C), suggesting an excellent stability. This work offers a unique idea of designing catalytic materials for industrial electrochemical water splitting.

## Supplementary Information

Below is the link to the electronic supplementary material.

Supplementary file1 (PDF 6653 KB)
