# Supplementary material for: Three-Phase Heterojunction NiMo-Based Nano-Needle for Water Splitting at Industrial Alkaline Condition
Source: Nanomicro Lett. 2021 Dec 9;14:20. doi: 10.1007/s40820-021-00744-x (PMC8660933; doi:10.1007/s40820-021-00744-x)
Supplement: Supplementary file 1 — Supplementary file1 (PDF 6653 KB) [file 40820_2021_744_MOESM1_ESM.pdf]

Supporting Information for

Three-Phase Heterojunction NiMo-Based Nano-Needle for Water Splitting at Industrial Alkaline Condition

Guangfu Qian[1], Jinli Chen[1], Tianqi Yu[1], Jiacheng Liu[1], Lin Luo[1], Shibin Yin[1,] *

[1]College of Chemistry and Chemical Engineering, State Key Laboratory of Processing for Non-Ferrous Metal and Featured Materials, Guangxi University, 100 Daxue Road, Nanning 530004, P. R. China

*Corresponding author. E-mail: yinshibin@gxu.edu.cn (S. Yin)

S1 Experimental Section

S1.1 Computational Methods

The Vienna Ab initio Software Package (VASP 5.3.5) code was used to obtain all the density functional theory (DFT) calculations under the projected augmented wave (PAW) approach and Perdew-Burke-Ernzerhof (PBE) generalized gradient approximation [S1-S4]. The cutoff energy was set at 450 eV for the plane-wave basis set. The Monkhorst-Pack (MP) grids were employed to optimize the Brillouin zone of the surface unit cell, and the k-point mesh density is 2π×0.04 $Å^{-1}$ [S5]. In order to complete the geometric optimization, the force and electronic self-consistent iteration were converged to 0.01 eV $Å^{-1}$ and 10^{-5} eV, respectively. For reducing the underestimation of the electronic band gap and the excessive tendency to delocalize the electron density, the electronic structure of catalysts was obtained by the PBE+U method. Herein, the Hubbard parameter of Ni and Mo were set to U−J=3 and 5 eV, respectively. To avoid interactions between periodic images, the vacuum layer was set to 15 Å.

S1.2 Preparation of Ni/MoO_2@CN Nano-needle

All reagents were produced by Aladdin Reagent Co., Ltd with no further purification. The 1.0 M HCl was used to remove the oxide on the surface of NF (1.0×2.0 cm^2) under the ultrasound condition, and following washed with ultra-pure water and ethanol for about 30 min. Then, the NF was immersed in a mixed solution [20 mL ethylene glycol, 5 mL ultra-pure water, 54 mg (0.186 mmol) $Ni(NO_3)_2·6H_2O$ and 210 mg (0.170 mmol) $(NH_4)_6Mo_7O_{24}·4H_2O$], which put into a 50 mL steel autoclave and maintained for 12 h at 140 °C. Subsequently, when the temperature cooled down to 25 °C, the NF was cleaned by C_2H_5OH and ultra-pure water, and vacuum dried at 80 °C for 12 h. Finally, it heated at different temperatures (350, 450, and 500 °C) for 2 h under the reducing atmosphere [5% H_2+95% Ar, named as (Ni-MoO_2)@CN nano-needle]. The mass loading of Ni/MoO_2@CN nano-needle is 15.2 mg cm^{-2} by ultrasonication method to remove the materials from NF. The samples with Ni/Mo molar ratios of 1:5 and 1:9 were prepared by the

same method. Besides, MoO_2@CN and Ni@CN were obtained by the same method without Ni and Mo source, respectively; Ni/MoO_2 was obtained in pure water solution with Mo and Ni source.

S1.3 Characterization

The SU8220 scanning electron microscopy (SEM, HITACHI, Japan) was employed to study the surface morphology of the samples. The G2 80-300 Titan ETEM (FEI Co., USA) worked at 300 kV to obtain the energy dispersive X-ray (EDX) spectroscopy and high-resolution transmission electron microscopy (HRTEM) images. The D8 Advance X-ray diffraction (XRD) with λ=0.15406 nm $Cu_{K\alpha}$ radiation (SmartLab, Rigaku Co., Japan) to research the crystal structure of catalysts. The state of elements for catalyst was obtained by the ESCALab 250Xi X-ray photoelectron spectroscopy (XPS, ThermoFisher Scientific, USA) with an Al X-ray source worked at 150 W. The Horiba Jobin Yvon Inc., France, $\lambda_{(He/Ne)}$=532 nm Raman spectrometer obtained the Raman spectroscopy.

S1.4 Electrochemical Measurements

Traditional three-electrode cell (include: all samples, reversible hydrogen electrode and graphite bar were used as work, reference and counter electrode, respectively) were used to evaluate linear sweep voltammetry (LSV), electrochemical impedance spectra (EIS) and chronopotentiometry (CP) for all catalysts, and obtained by electrochemical workstation (Germany) under 1.0 M KOH+30 °C solution containing saturated N_2. EIS was evaluated at −0.2 and 1.5 V for HER and OER with the range from 100,000 to 0.1 Hz and the amplitude is 5 mV. The *iR* correction potential (E_{corr}) was obtained by the following equation: (1) $E_{corr}=E_{mea}-iR_s$, the actually measured potential and solution resistance were the E_{mea} and R_s. The WS performance was tested by the two-electrode cell at the same environment. The Tafel plots were originated from LSV curves by the formula: [(2) $\eta=b\log|j|+a$], the current density, intercept and Tafel slope are *j*, *a* and *b*, respectively.

Furthermore, the cathode/anode noble metal ink contained 40 wt% IrO_2/C and 20 wt% Pt/C (purchased from Aladdin without further purification), which dispersed in a mixed solution [5.0 wt% Nafion (40.0 μL) and ethanol (0.96 mL)]. Subsequently, it was spread on the 0.5 cm^2 NF (named as IrO_2/C and Pt/C).

S2 Supplementary Figures

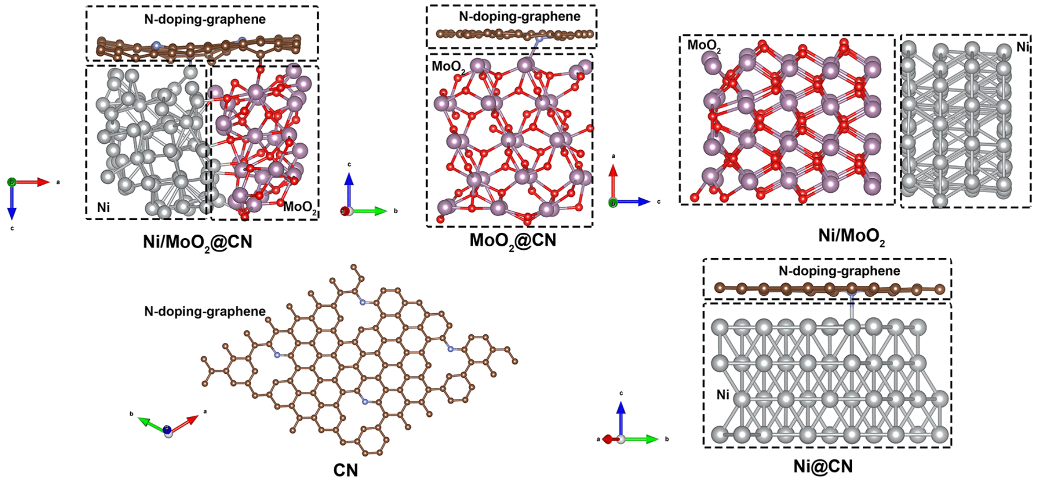

Fig. S1 Theoretical structure models of Ni/MoO_2@CN, MoO_2@CN, Ni/MoO_2, CN and Ni@CN

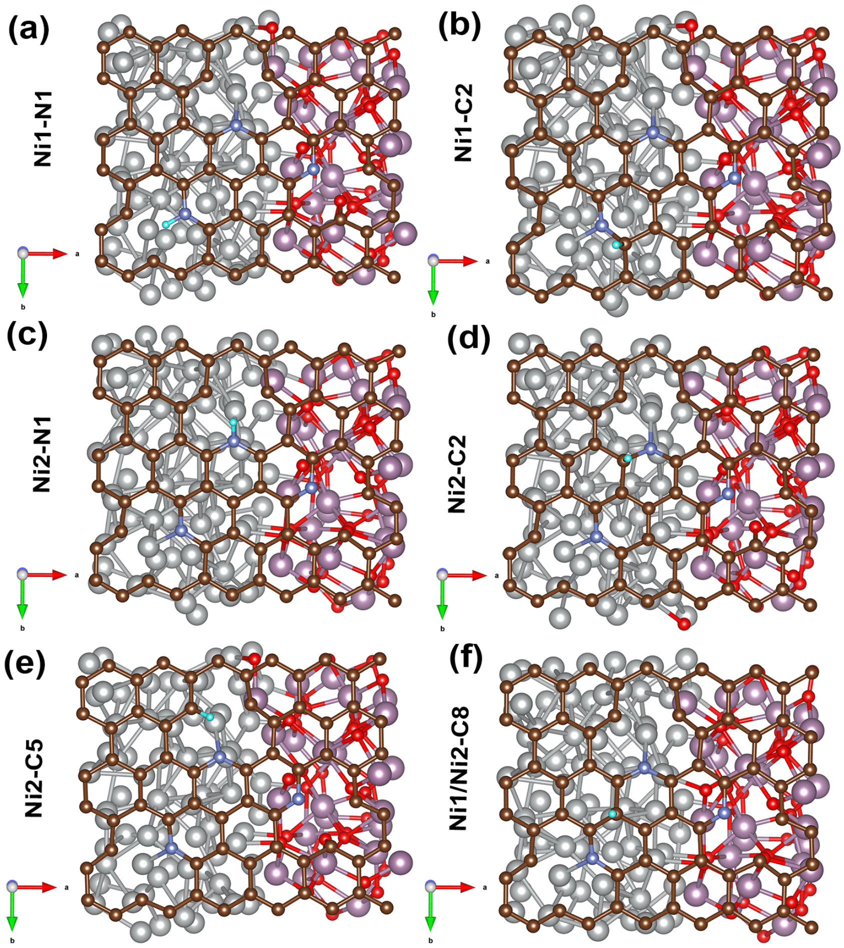

Fig. S2 Schematic illustration of H adsorption for Ni/MoO_2@CN model

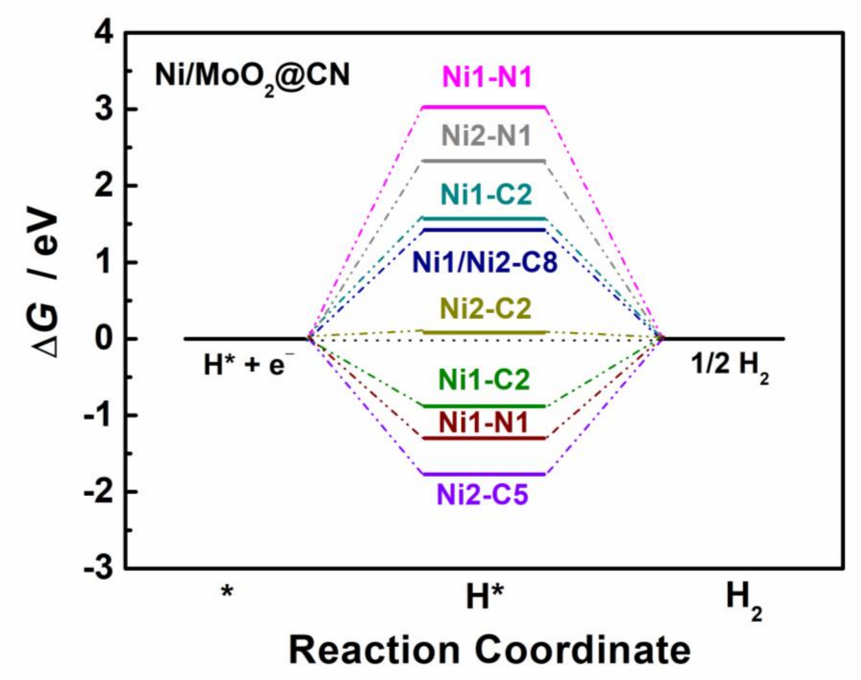

Fig. S3 ΔG_{H*} calculated at different adsorb sites for Ni/MoO_2@CN model

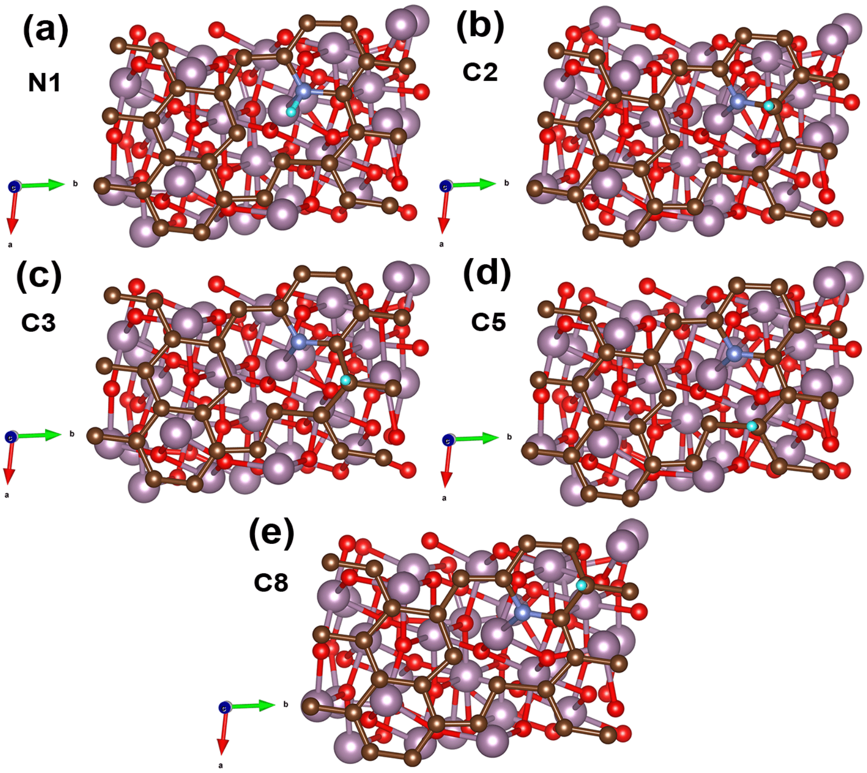

Fig. S4 Schematic illustration of H adsorption for MoO_2@CN model

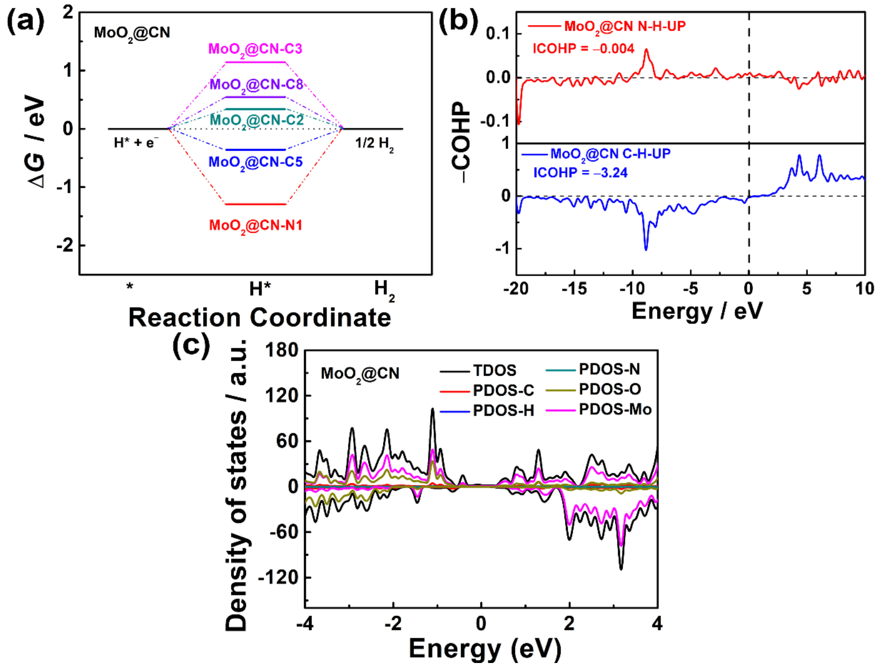

Fig. S5 (**a**) ΔG_{H^*} calculated at different adsorb sites for MoO_2@CN model; (**b**) COHP and (**c**) PDOS analysis for MoO_2@CN model with the H atom adsorbed on the sites

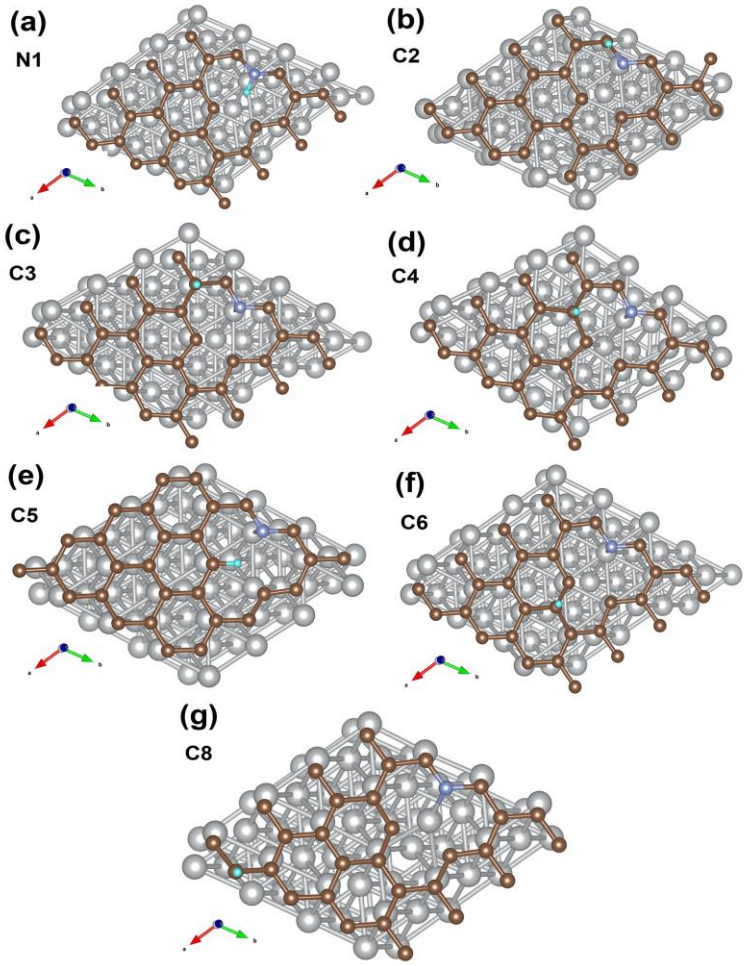

Fig. S6 Schematic illustration of H adsorption for Ni@CN model

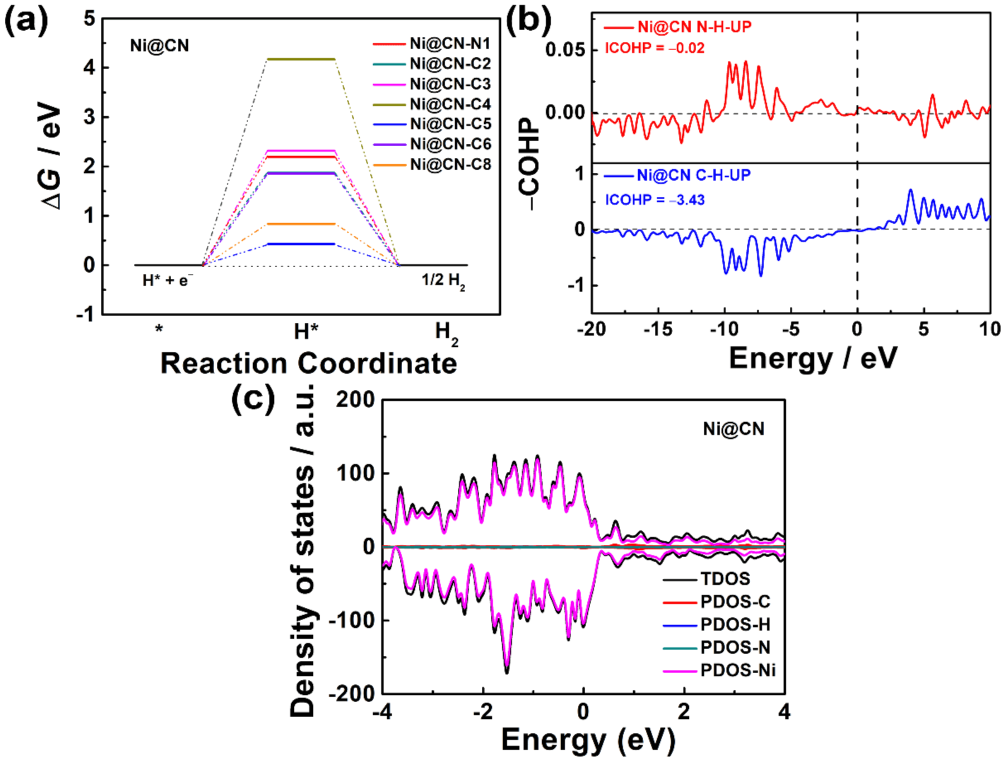

Fig. S7 (**a**) ΔG_{H*} calculated at different adsorb sites for Ni@CN model; (**b**) COHP and (**c**) PDOS analysis for Ni@CN model with the H atom adsorbed on the sites

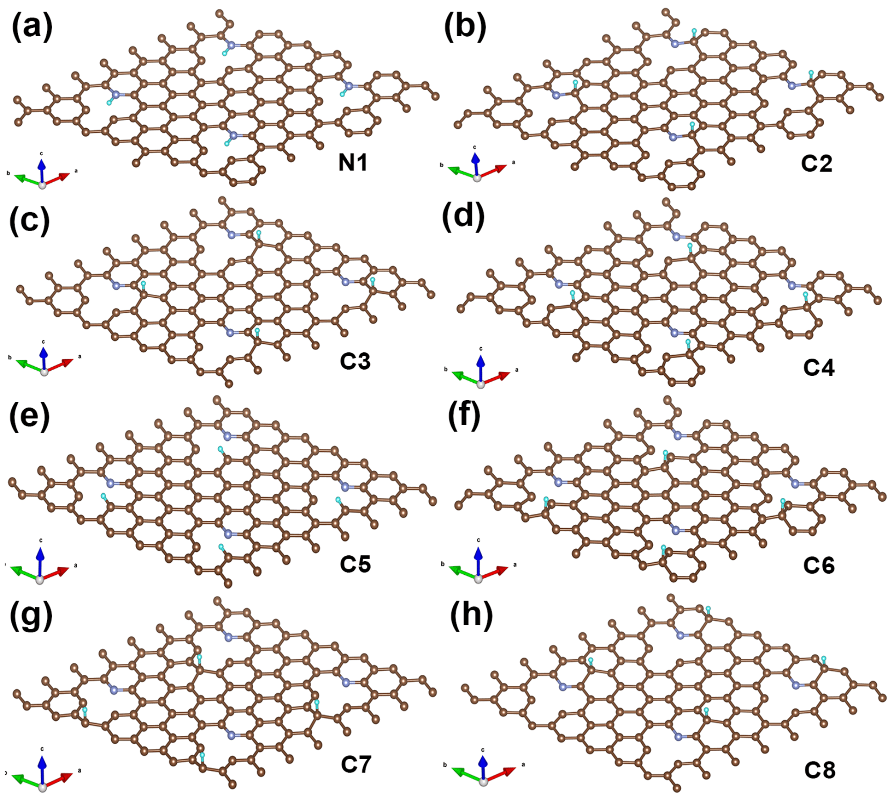

Fig. S8 Schematic illustration of H adsorption for CN model

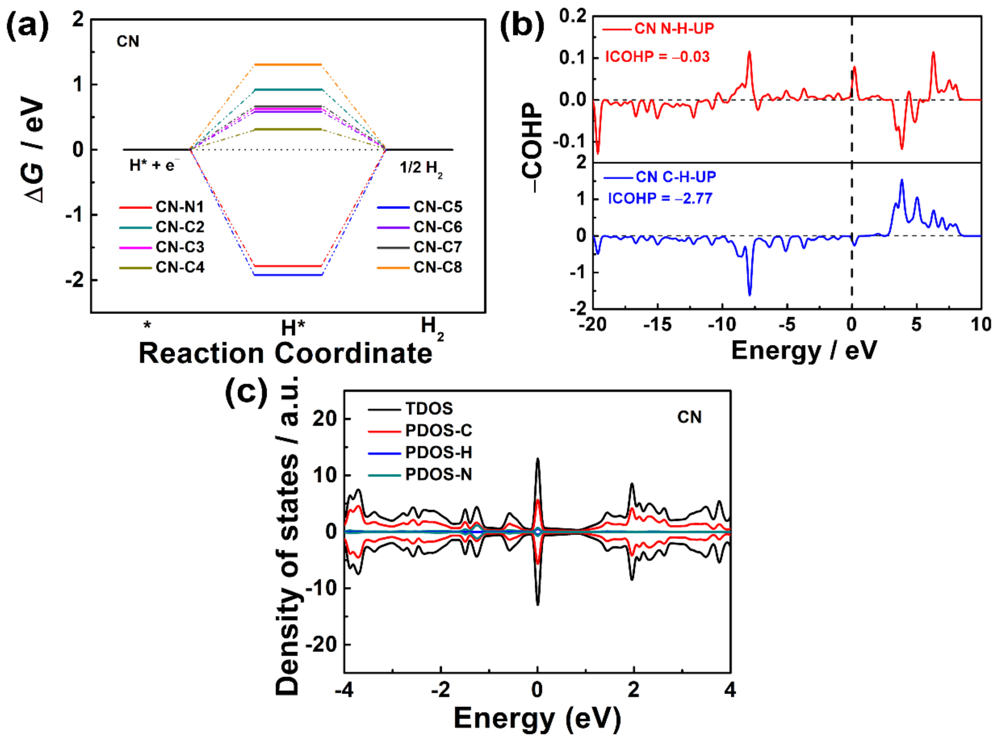

Fig. S9 (**a**) ΔG_{H*} calculated at different adsorb sites for CN model; (**b**) COHP and (**c**) PDOS analysis for CN model with the H atom adsorbed on the sites

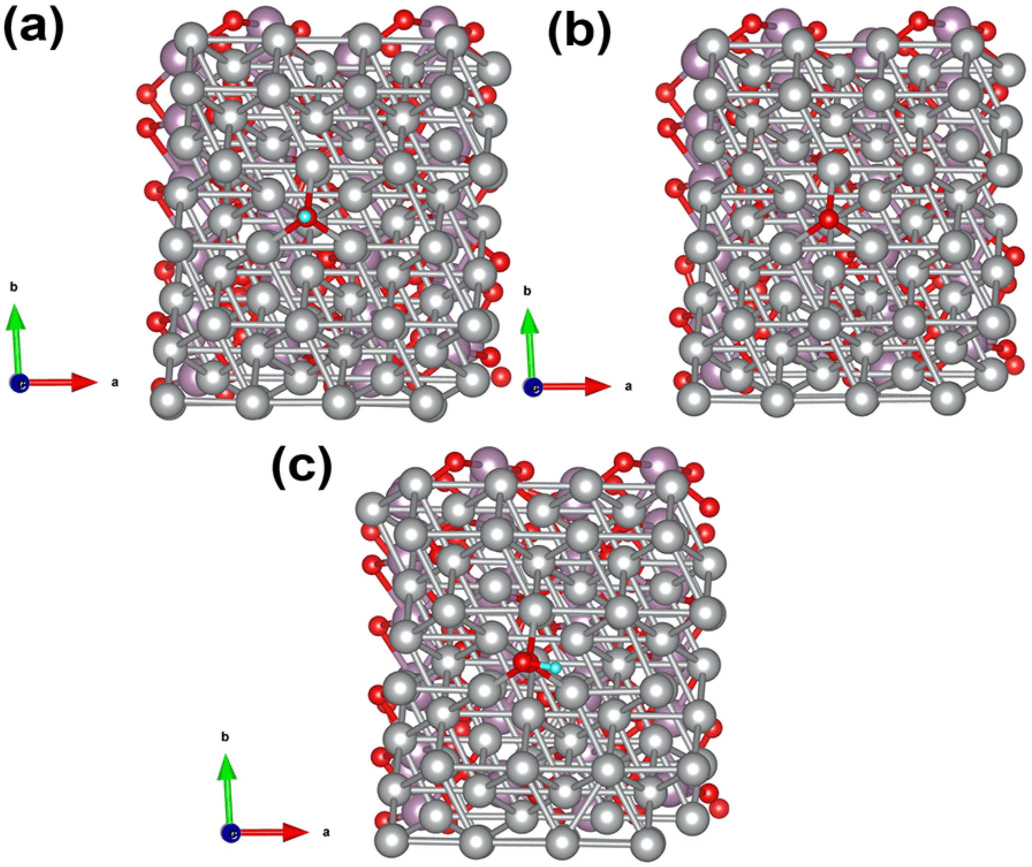

Fig. S10 Schematic illustration of *OH, *O and *OOH adsorption for Ni/MoO_2 model

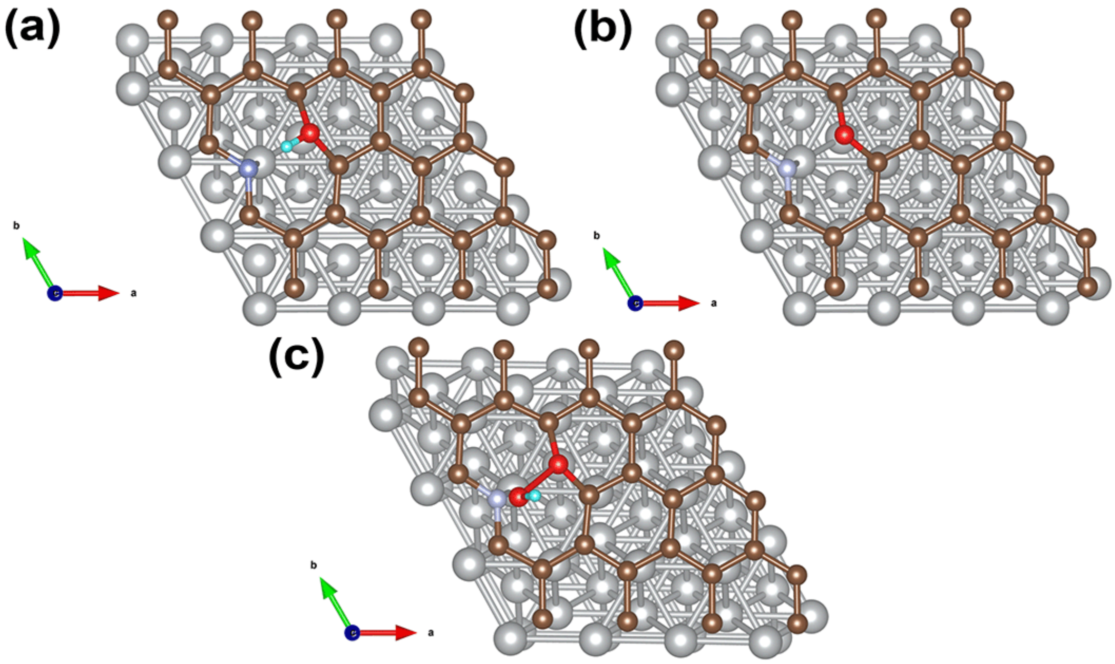

Fig. S11 Schematic illustration of *OH, *O and *OOH adsorption for Ni@CN model

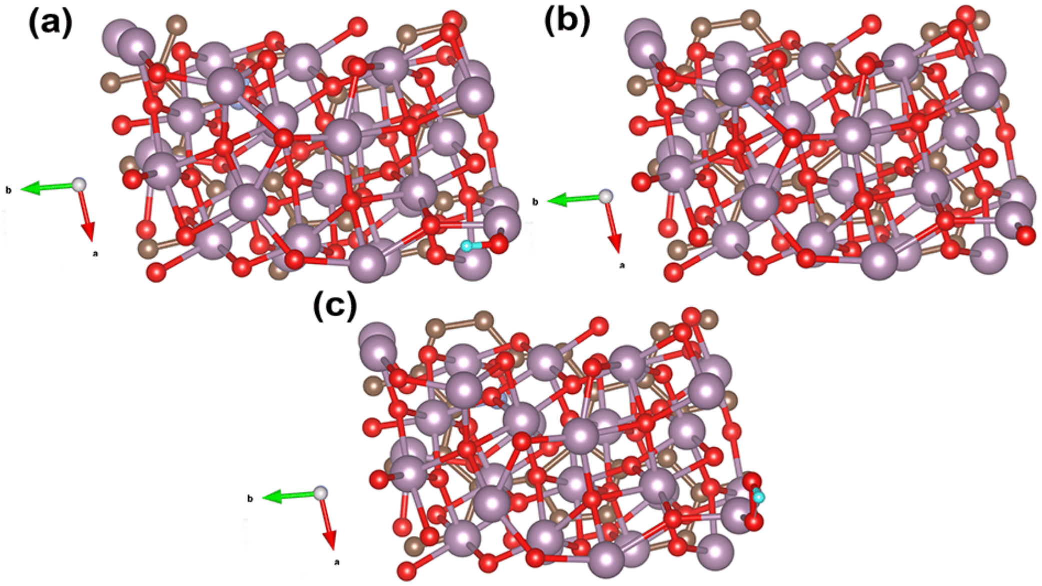

Fig. S12 Schematic illustration of *OH, *O and *OOH adsorption for MoO_2@CN model

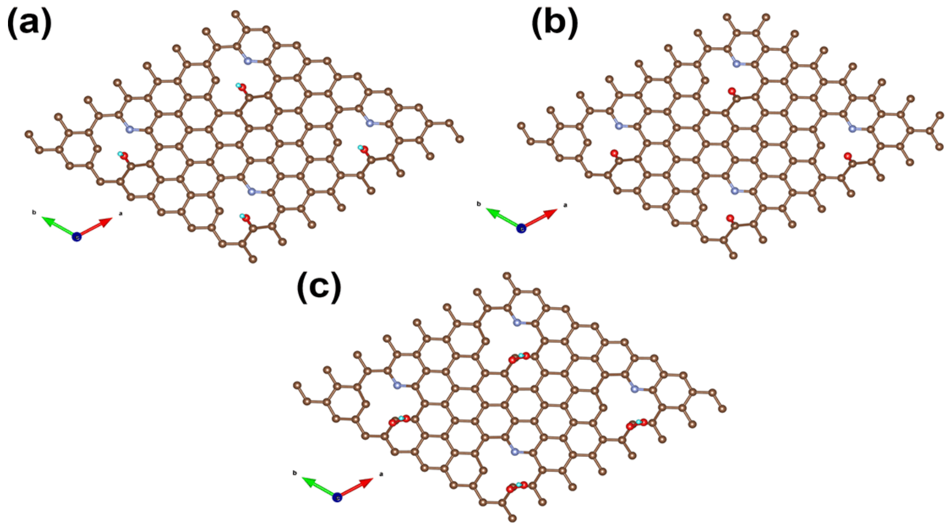

Fig. S13 Schematic illustration of *OH, *O and *OOH adsorption for CN model

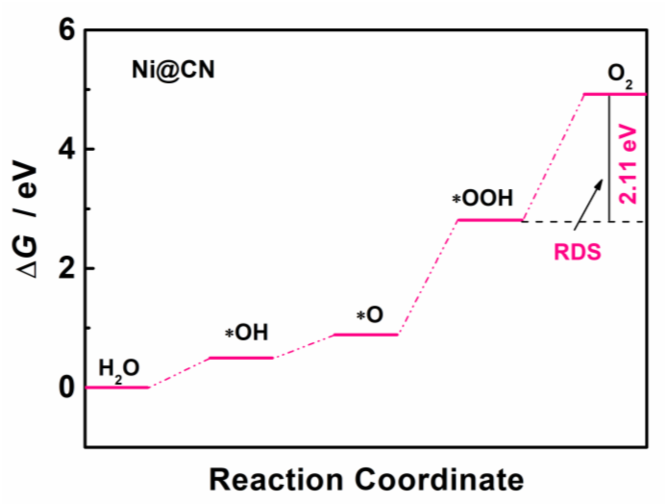

Fig. S14 OER reaction pathway for Ni@CN model

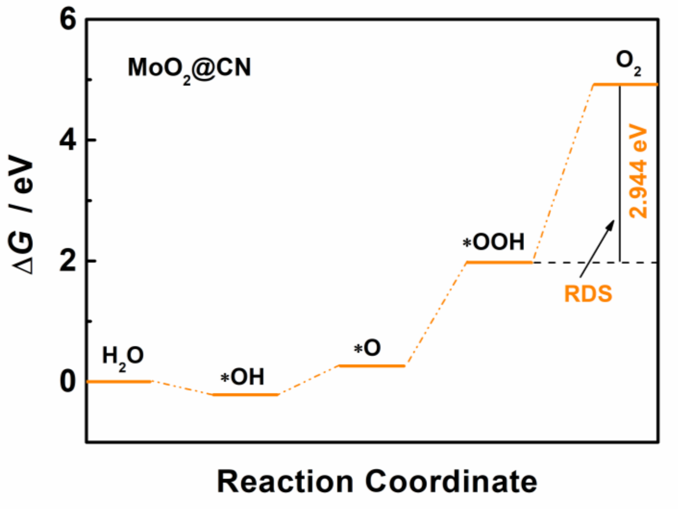

Fig. S15 OER reaction pathway for MoO_2@CN model

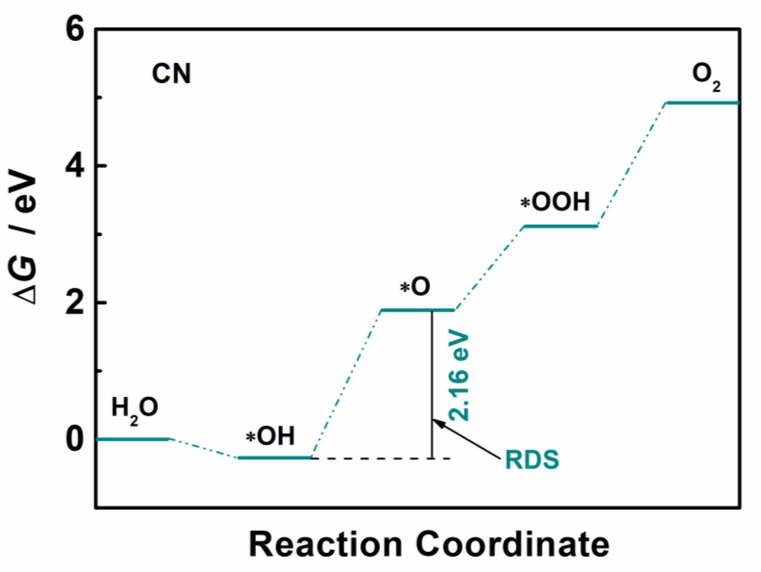

Fig. S16 OER reaction pathway for CN model

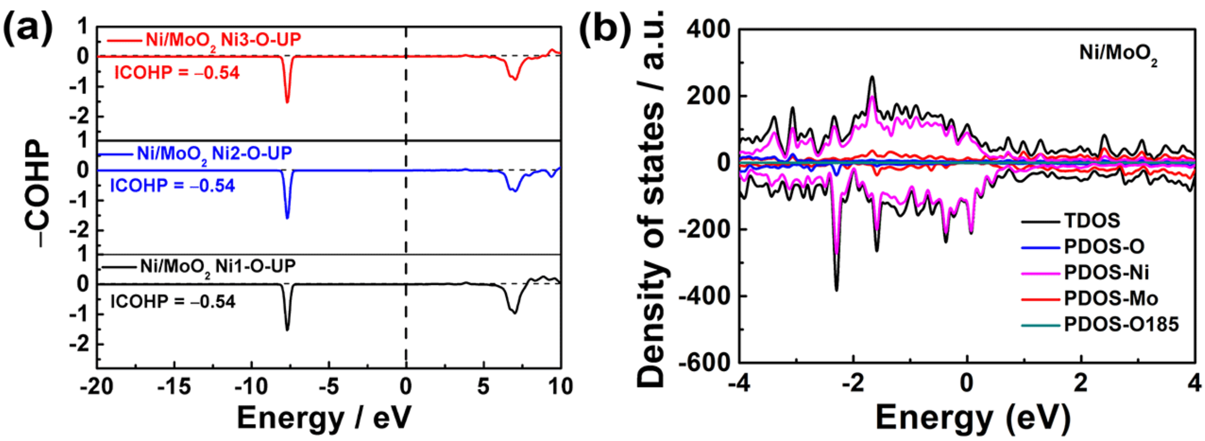

Fig. S17 (**a**) COHP and (**b**) PDOS analysis for the Ni/MoO_2 model with the O atom adsorbed on the sites

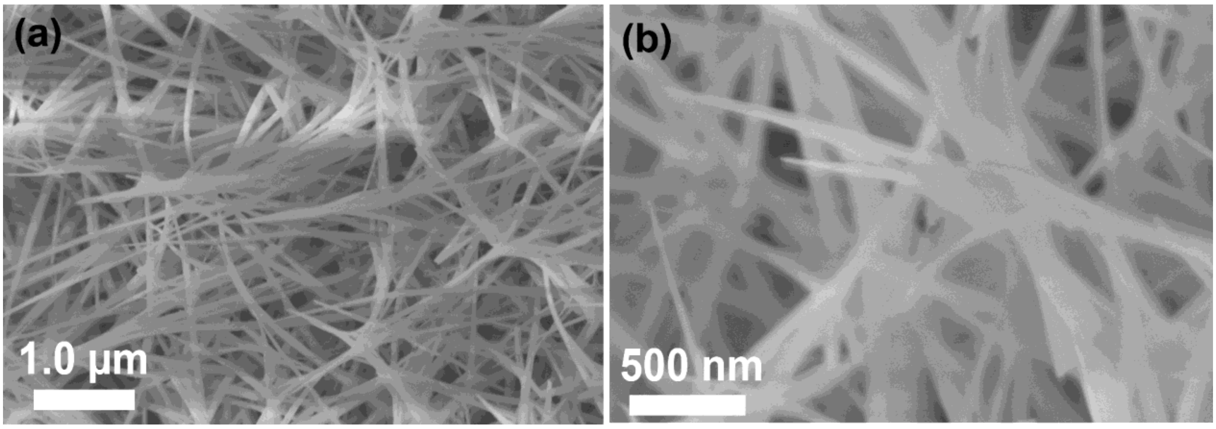

Fig. S18 SEM images of precursor

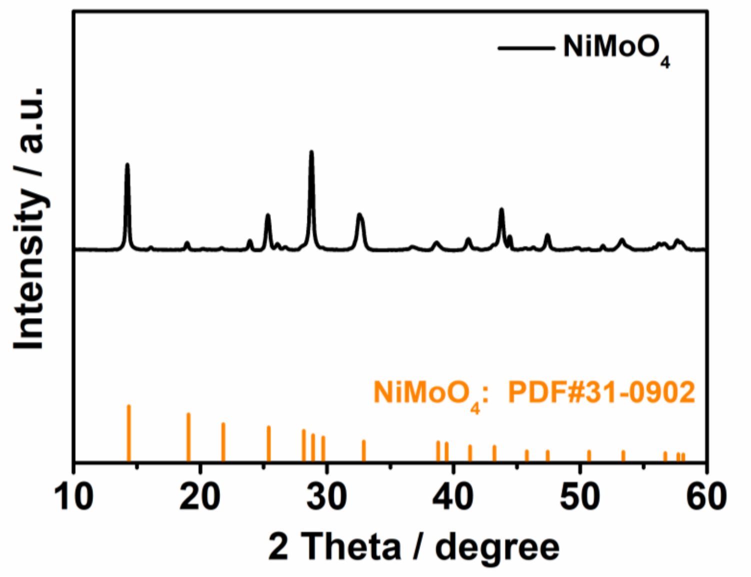

Fig. S19 XRD spectrum of precursor

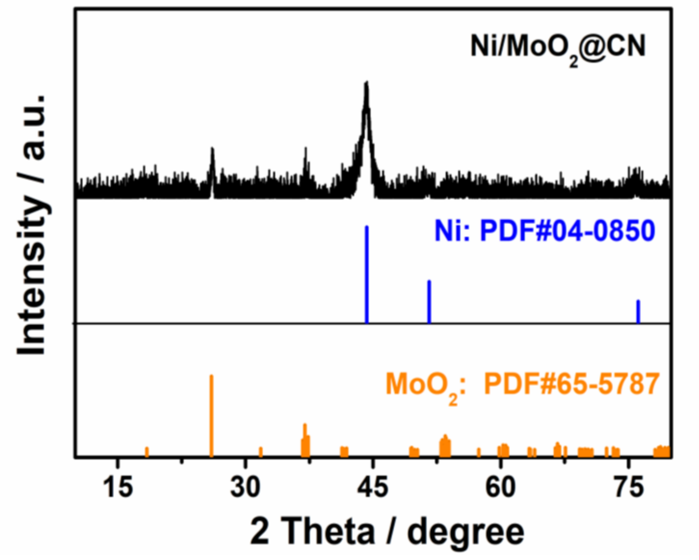

Fig. S20 XRD spectrum of Ni/MoO_2@CN

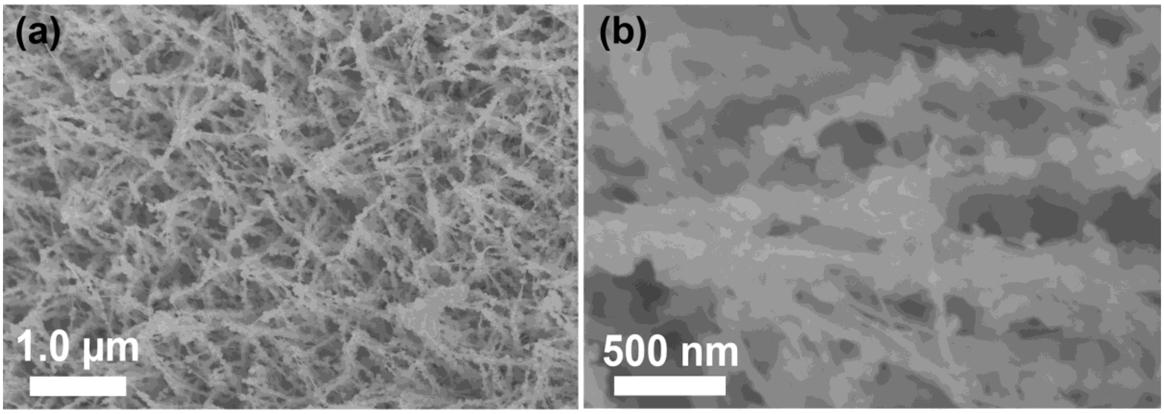

Fig. S21 SEM images of Ni/MoO_2@CN

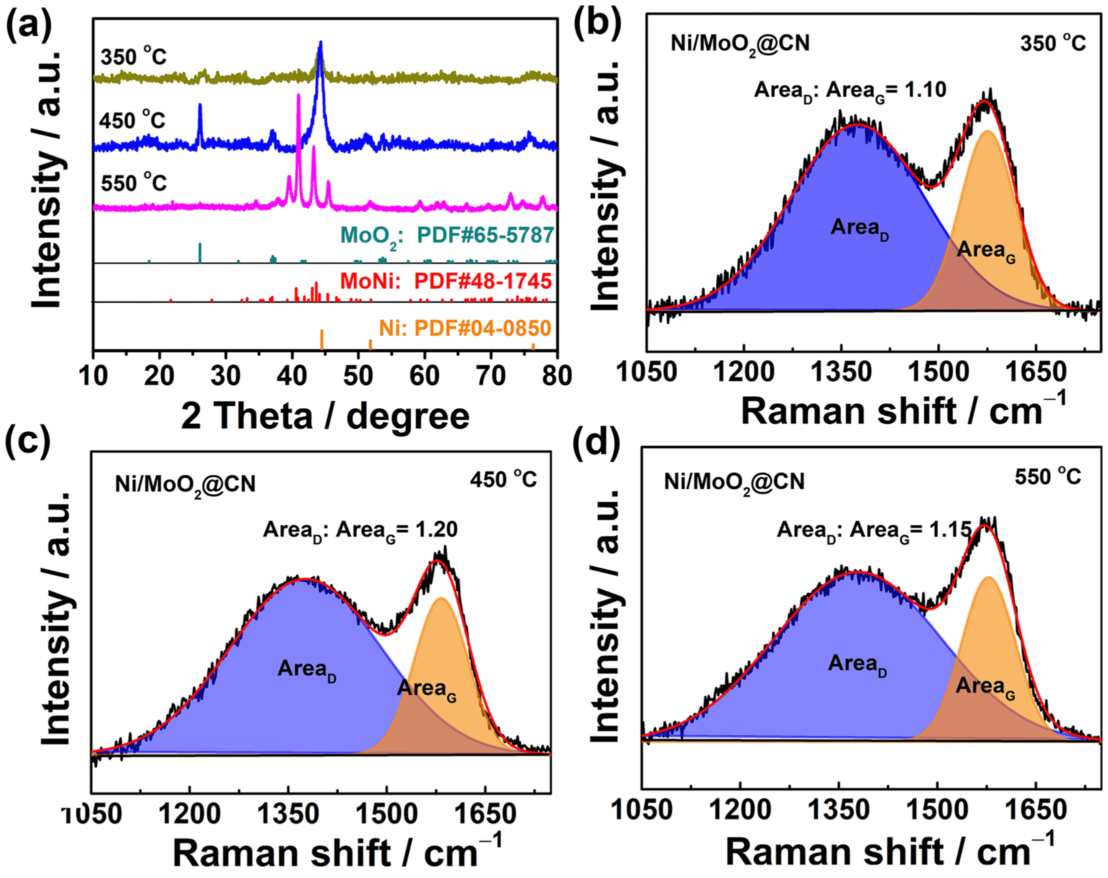

Fig. S22 (**a**) XRD and Raman spectra of Ni/MoO2@CN obtained at (**b**) 350 °C, (**c**) 450 °C and (**d**) 550 °C

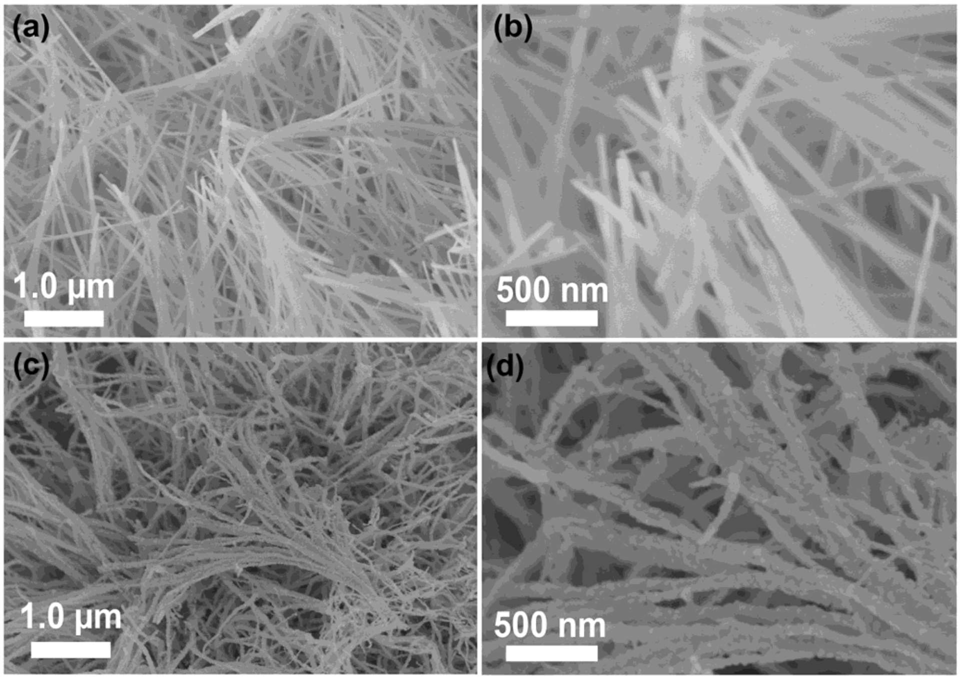

Fig. S23 SEM images of Ni/MoO2@CN obtained at (**a, b**) 350 °C and (**c, d**) 550 °C

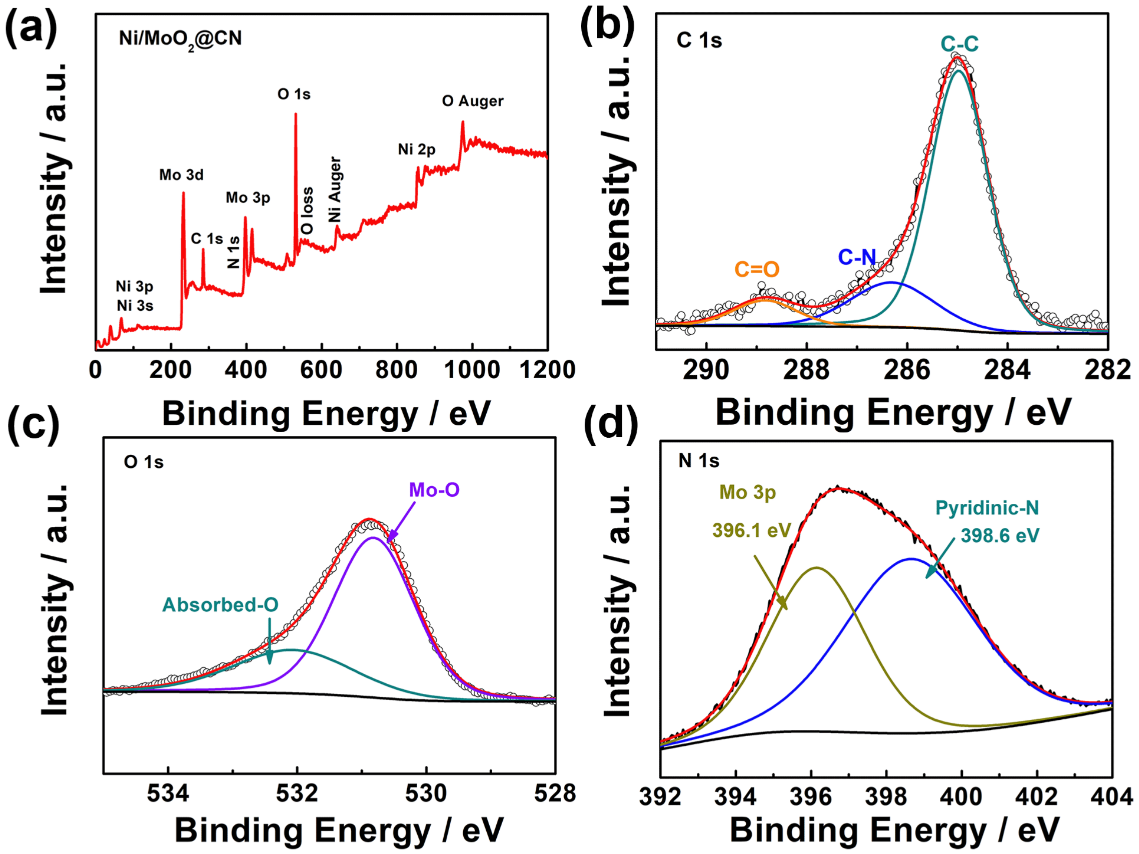

Fig. S24 (**a**) XPS summary, (**b**) C 1s, (**c**) O 1s and (**d**) N 1s spectra for Ni/MoO_2@CN

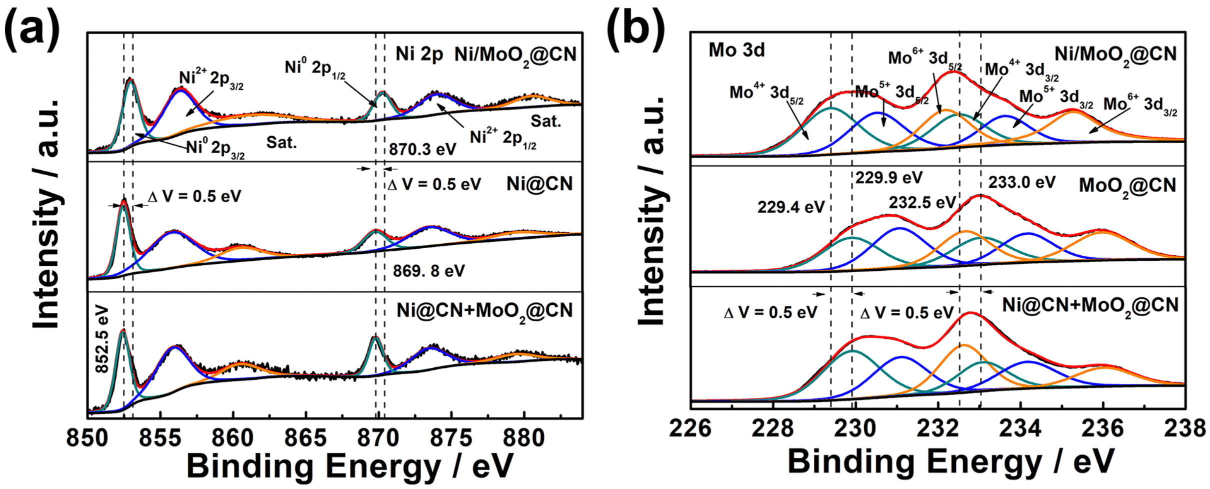

Fig. S25 HRXPS spectra of (**a**) Ni 2p and (**b**) Mo 3d for Ni/MoO_2@CN, MoO_2@CN, Ni@CN and Ni@CN+MoO_2@CN hybrids

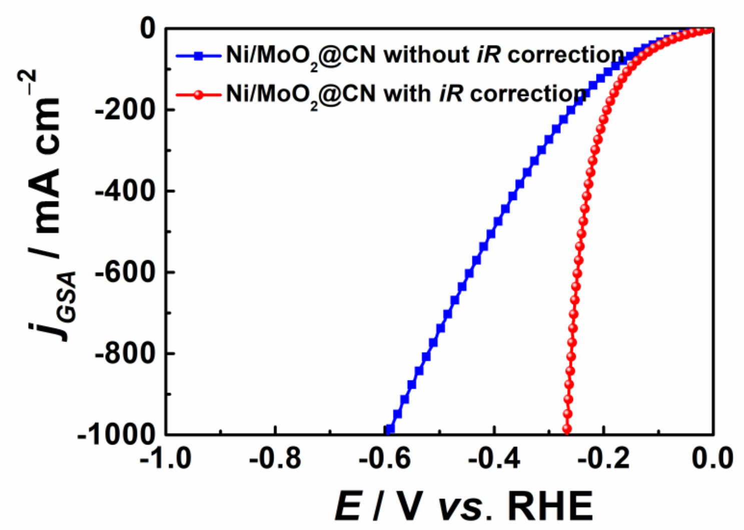

Fig. S26 HER LSV curves of Ni/MoO_2@CN with/without *iR* correction

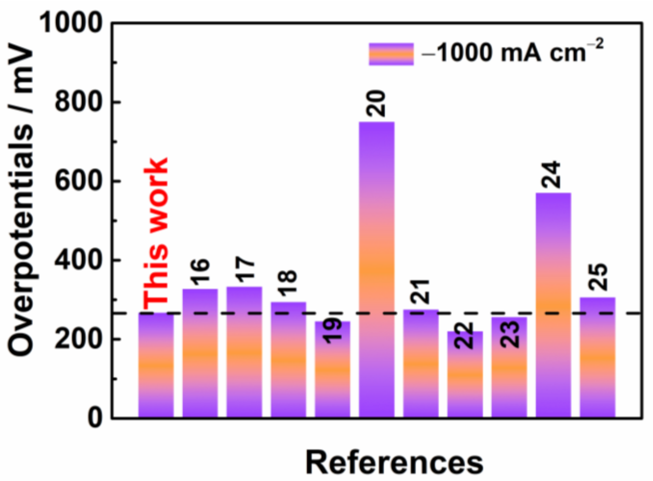

Fig. S27 Comparisons of HER activity of Ni/MoO_2@CN with other reported non-noble-metal catalysts

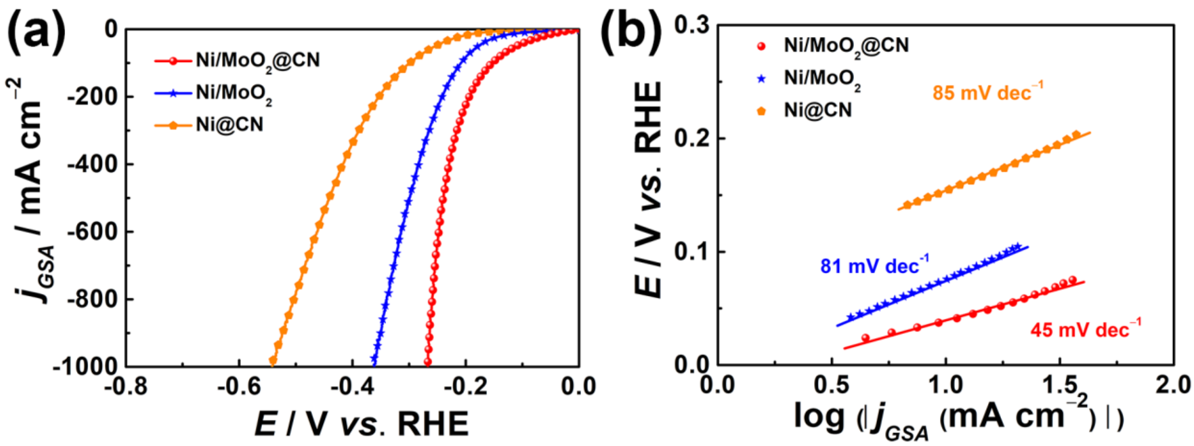

Fig. S28 (**a**) HER LSV curves and (**b**) corresponding Tafel plots of HER for investigated samples

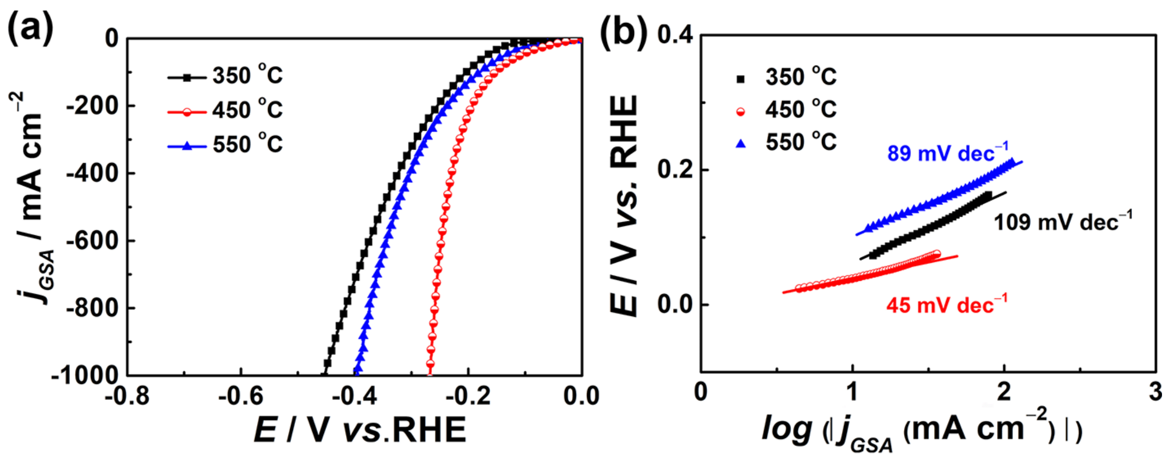

Fig. S29 (**a**) LSV curves and (**b**) Tafel slopes of HER for precursors annealed at different temperatures

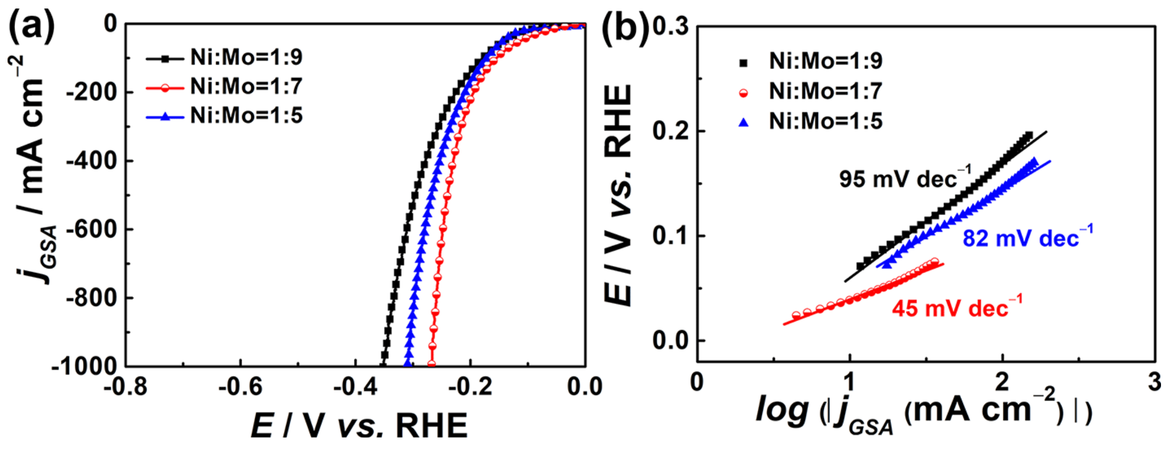

Fig. S30 (**a**) LSV curves and (**b**) Tafel slopes of HER with different Ni/Mo molar ratios

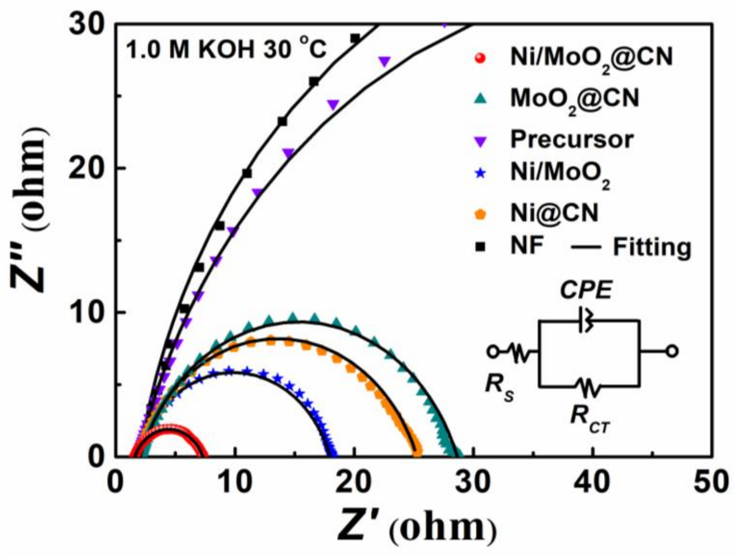

Fig. S31 Nyquist plots tested at −0.2 V for HER with a frequency from 100,000 to 0.1 Hz in 1.0 M KOH; Inset is the equivalent circuit model

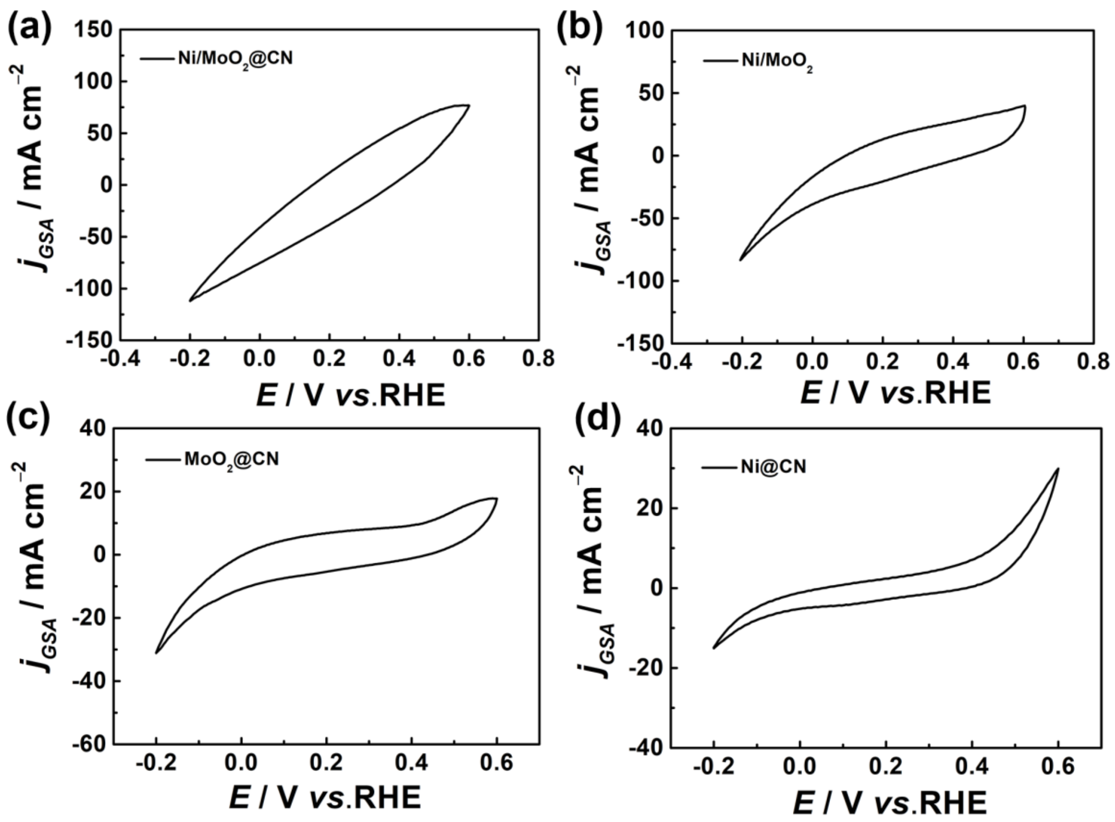

Fig. S32 CV curves of Ni/MoO_2@CN, Ni/MoO_2, MoO_2@CN and Ni@CN in 1.0 M PBS (pH=6.87) with a scan rate of 50 mV s^{-1}

We used the CV method to study the TOF of Ni/MoO_2@CN, Ni/MoO_2, Ni@CN and MoO_2@CN for HER [S6-S10]. As shown in **Fig. S32**, the Ni/MoO_2@CN, Ni/MoO_2, Ni@CN and MoO_2@CN are tested in 1.0 M phosphate buffer solution (PBS, pH=6.87), and the region is −0.2 to 0.6 V *vs*. RHE. The total number of active atoms should be proportional to the potential region range.

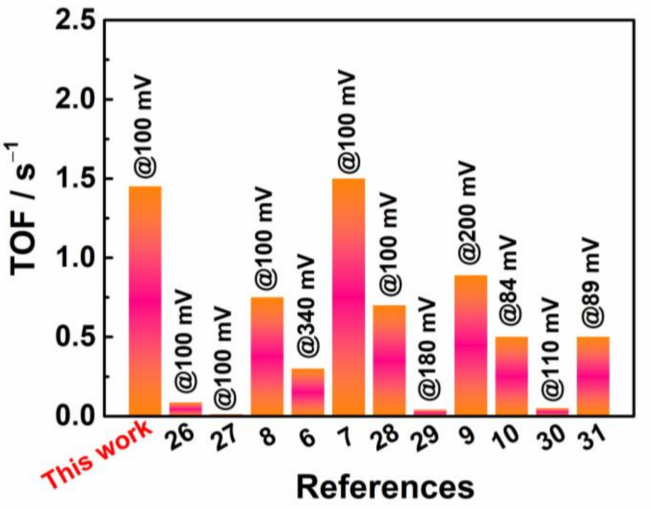

Fig. S33 Comparisons of TOF values of Ni/MoO_2@CN for HER with other reported non-noble-metal catalysts

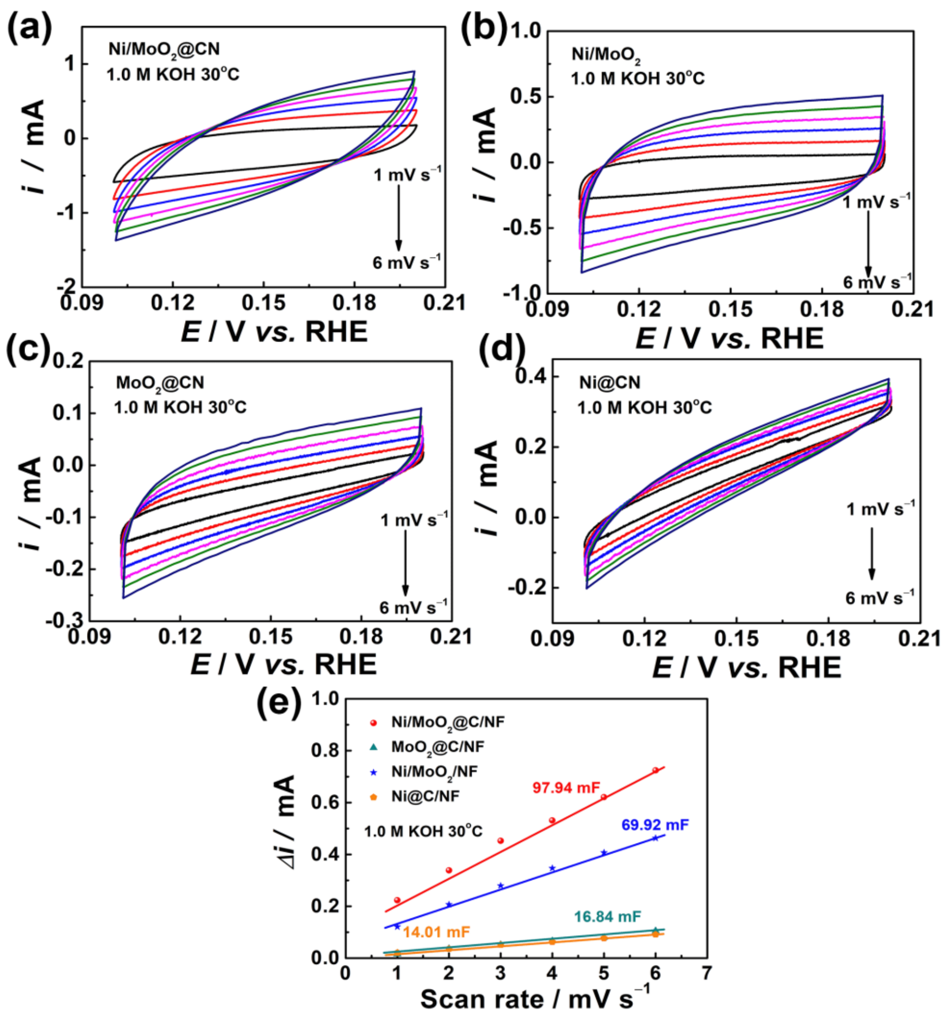

Fig. S34 (**a-d**) Typical CV curves of the samples with scan rates ranging from 1 to 6 mV s^{-1}, the scanning potential range is from 0.10 V to 0.20 V; (**e**) Estimation of C_{dl} by plotting the capacitive current density against the scan rate to fit a linear regression

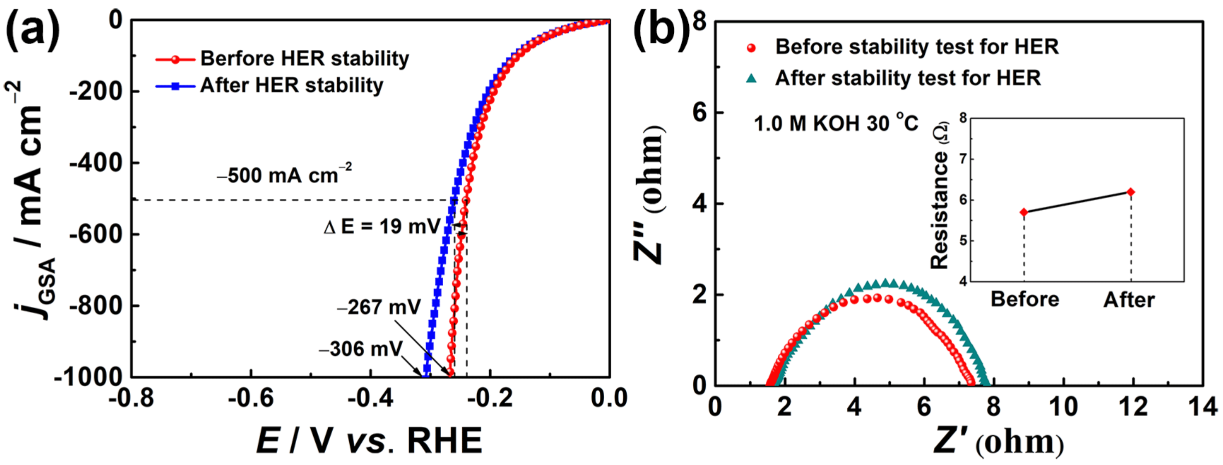

Fig. S35 (**a**) HER LSV curves and (**b**) R_{ct} of Ni/MoO_2@CN before and after HER stability test

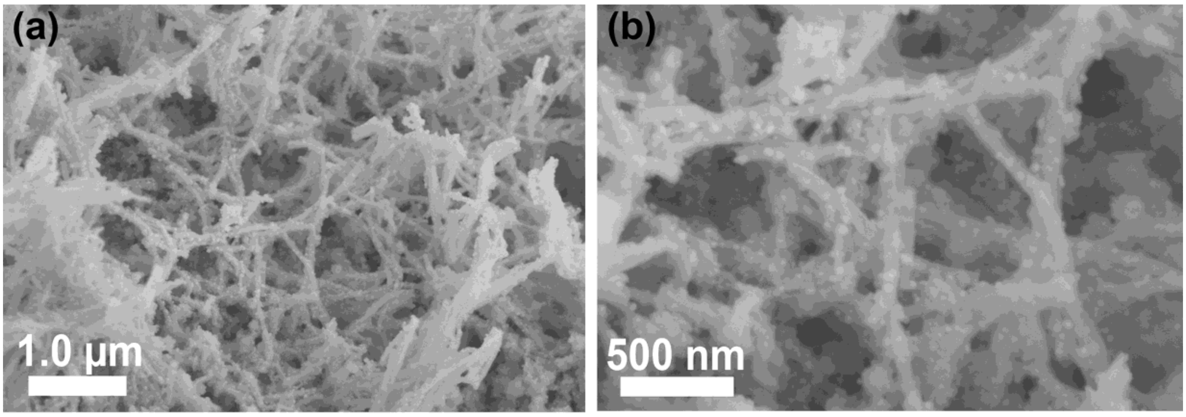

Fig. S36 SEM images of Ni/MoO_2@CN after HER stability test

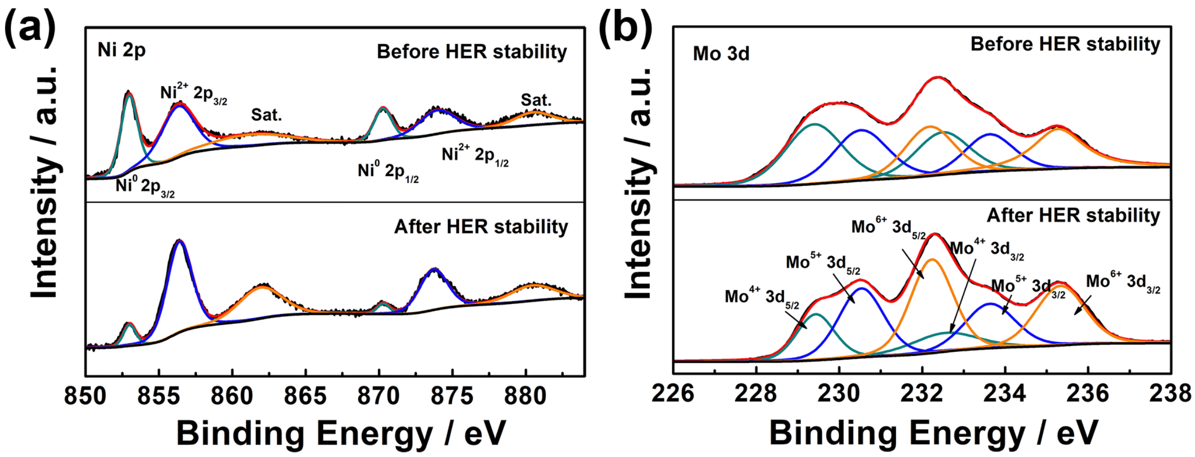

Fig. S37 XPS spectra of Ni/MoO_2@CN before and after HER stability test

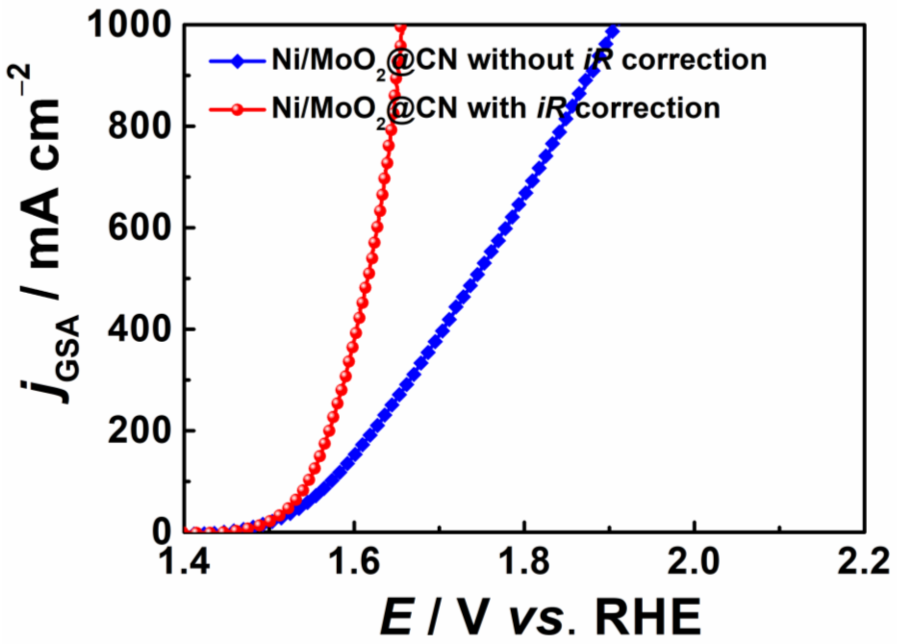

Fig. S38 OER LSV curves of Ni/MoO_2@CN with/without *iR* correction

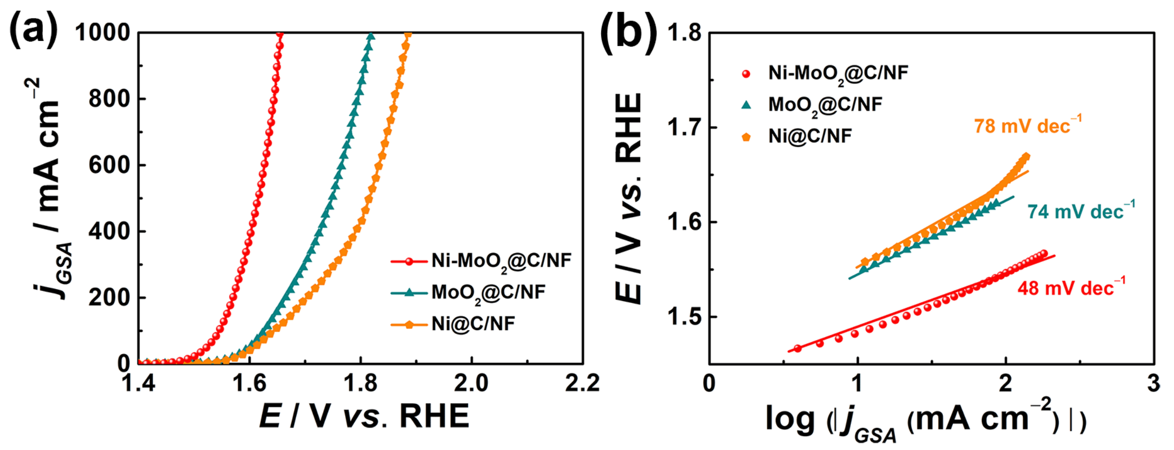

Fig. S39 (**a**) OER LSV curves and (**b**) corresponding Tafel plots of OER for investigated samples

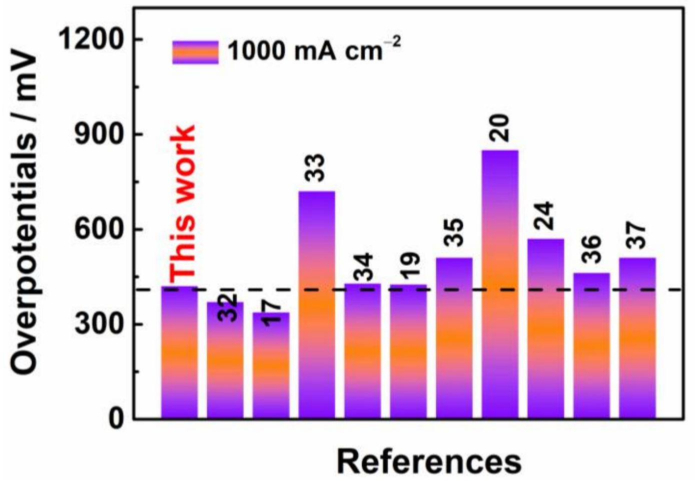

Fig. S40 Comparisons of OER activity of Ni/MoO_2@CN with other reported non-noble-metal catalysts

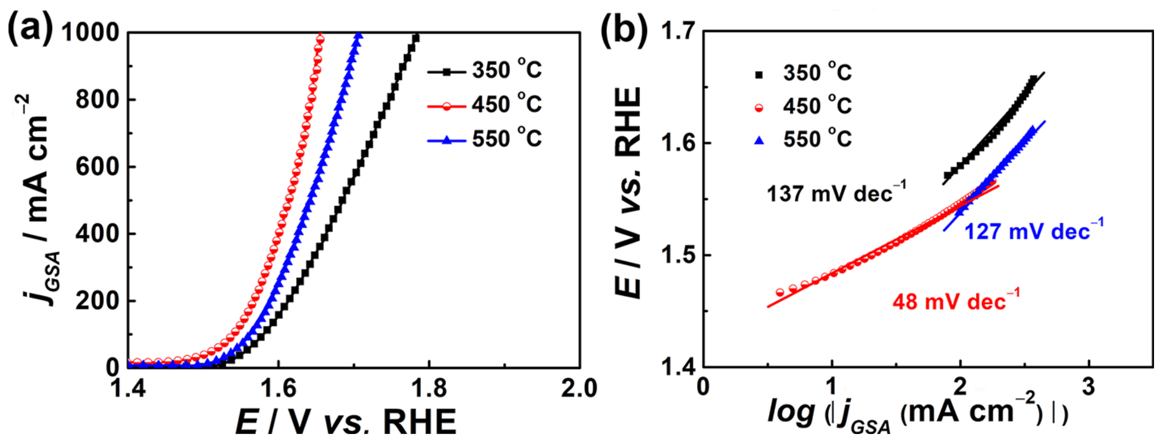

Fig. S41 (**a**) LSV curves and (**b**) Tafel slopes of OER for precursors annealed at different temperatures

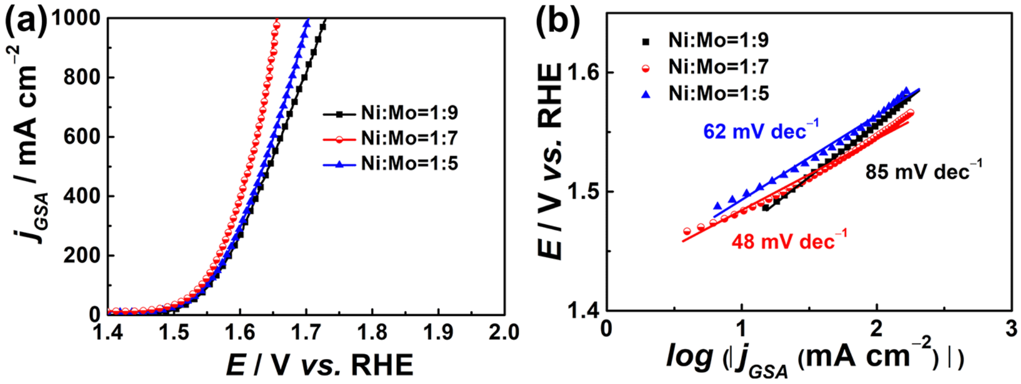

Fig. S42 (**a**) LSV curves and (**b**) Tafel slopes of OER with different Ni/Mo molar ratios

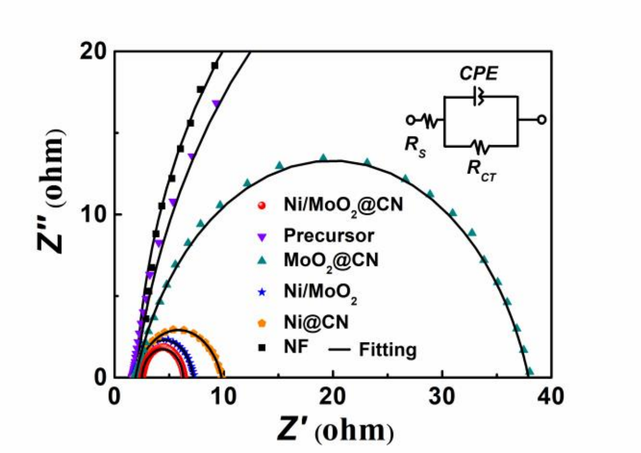

Fig. S43 Nyquist plots tested at 1.5 V for OER with a frequency from 100,000 to 0.1 Hz in 1.0 M KOH; Inset is the equivalent circuit model

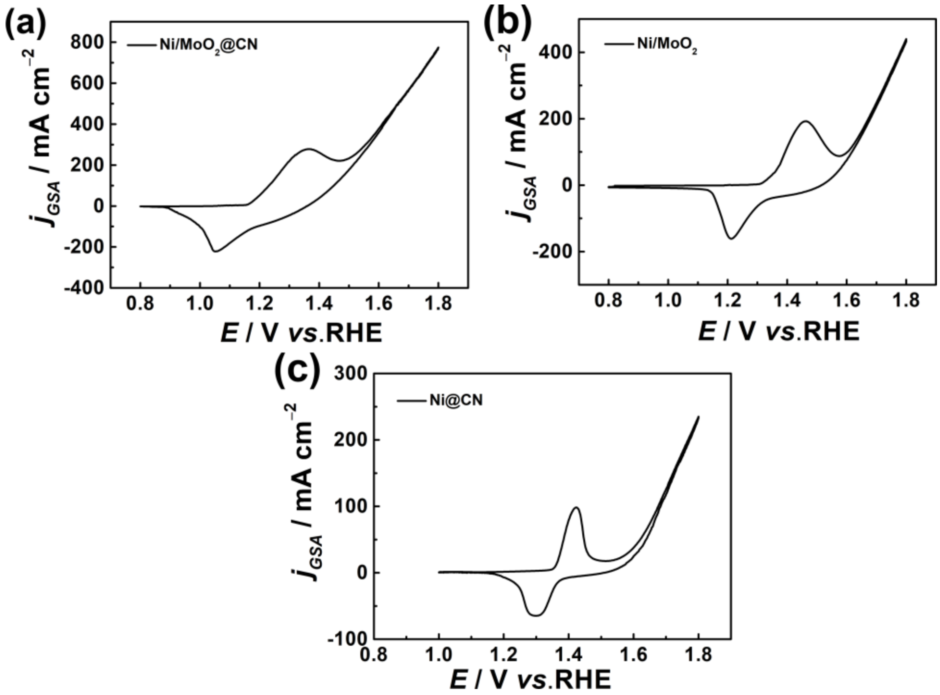

Fig. S44 CV curves of Ni/MoO_2@CN, Ni/MoO_2 and Ni@CN for determining the redox surface sites of Ni^{2+}/Ni^{3+} in 1.0 M KOH with a scan rate of 50 mV s^{-1}

We used the active surface redox sites method to study the TOFs of Ni/MoO_2@CN, Ni/MoO_2, and Ni@CN for OER, by calculating the redox surface sites of Ni^{2+}/Ni^{3+} without the capacitive current [S7, S11-S15]. As shown in **Fig. S44**, the Ni/MoO_2@CN, Ni/MoO_2 and Ni@CN are tested in 1.0 M KOH solution and the region is 1.0 to 1.8 V *vs*. RHE. The total number of active atoms is equal to the calculated charge of the peak Q_s divided by the charge of an electron (1.6×10^{-19} C), and the formula is $N_s=Q_s/Q_e$, which is from the one-electron reaction of Ni^{2+}/Ni^{3+}.

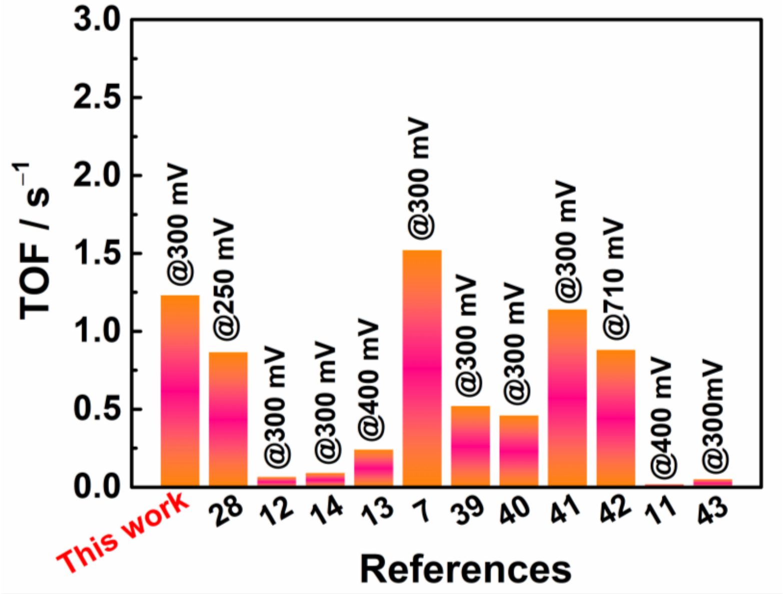

Fig. S45 Comparison of TOF values of Ni/MoO_2@CN for OER with other reported non-noble-metal catalysts

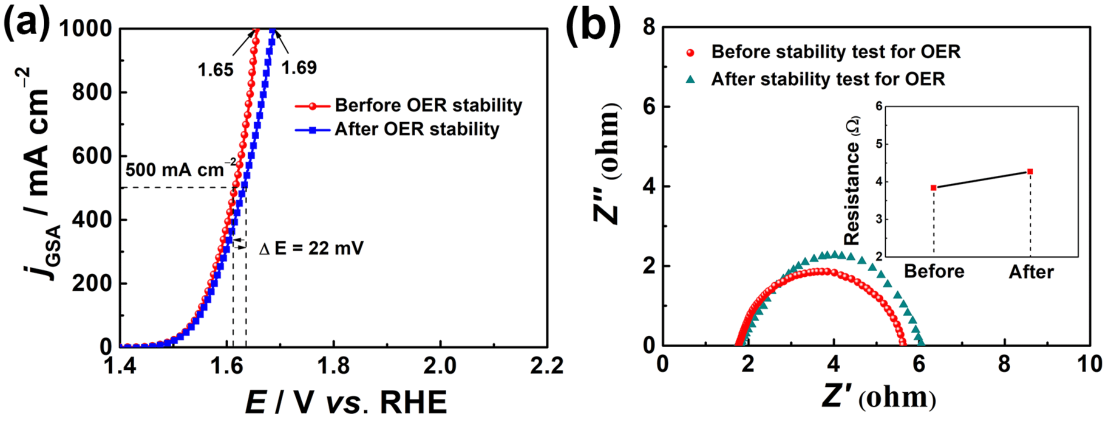

Fig. S46 (**a**) LSV curves and (**b**) R_{ct} of Ni/MoO_2@CN before and after OER stability test

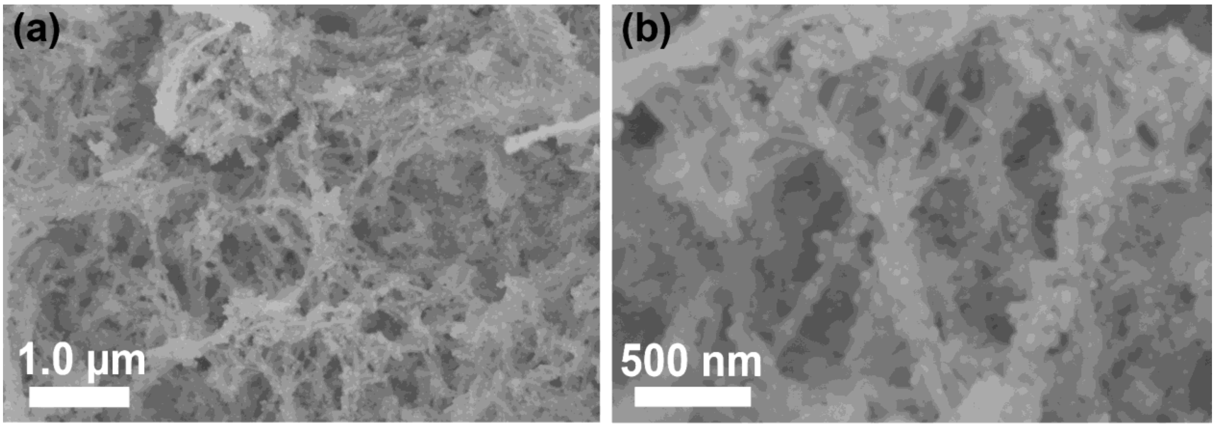

Fig. S47 SEM images of Ni/MoO_2@CN after OER stability test

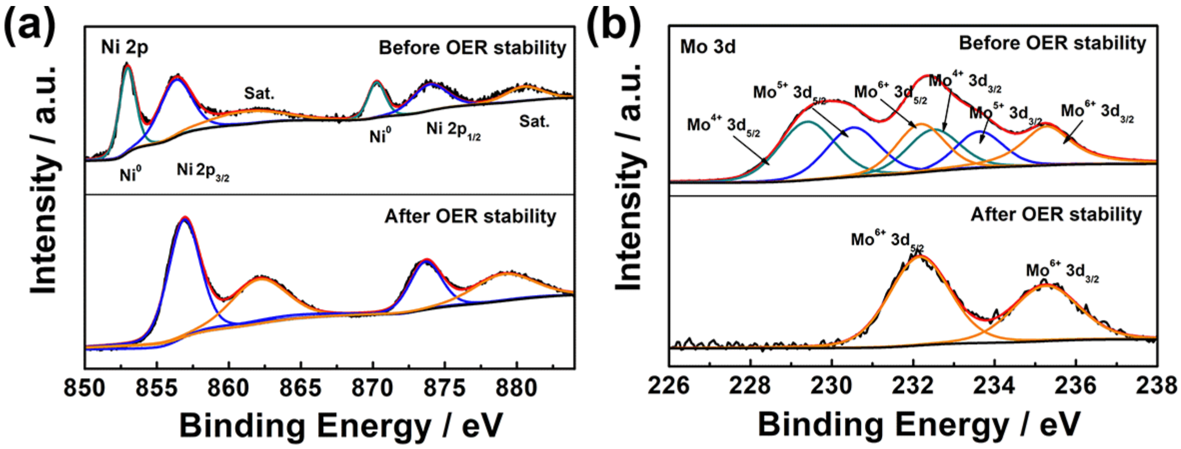

Fig. S48 XPS spectra of Ni/MoO_2@CN before and after OER stability test

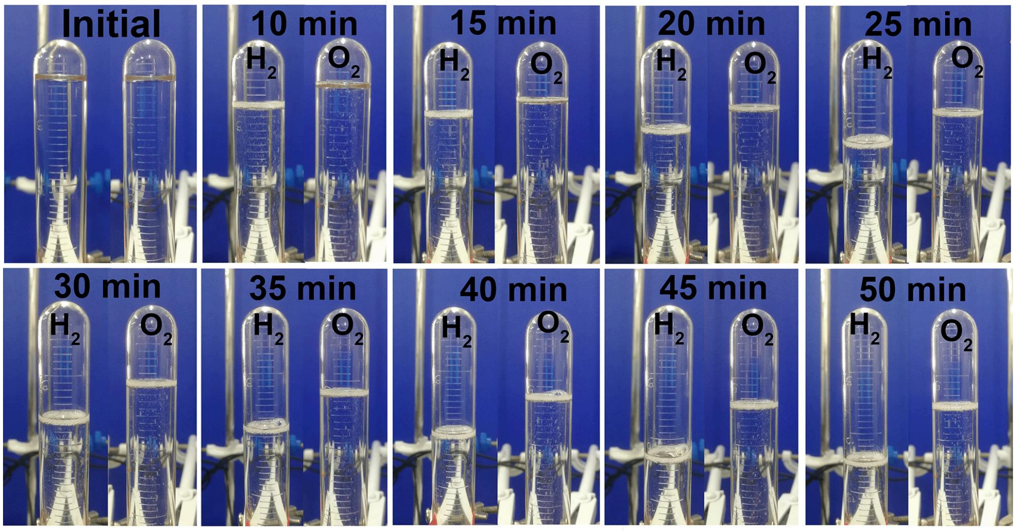

Fig. S49 Volume of H_2 and O_2 actually measured at 30.0 mA versus time for Ni/MoO_2@CN in 1.0 M KOH solution

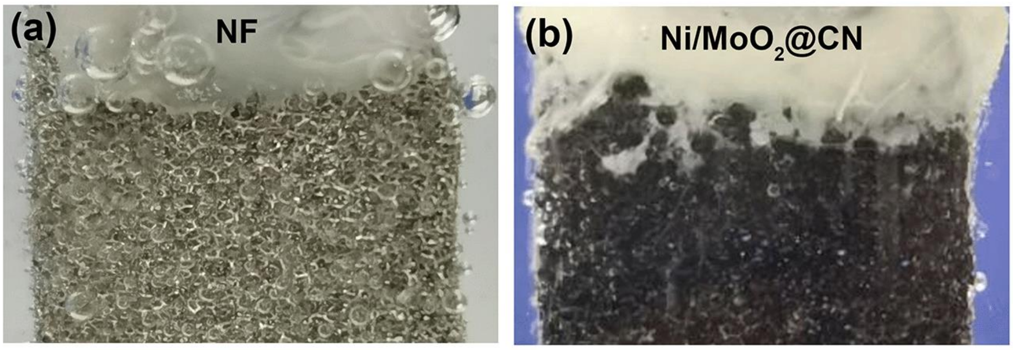

Fig. S50 Digital images of the generated H_2 bubbles on (a) NF and (b) Ni/MoO_2@CN.

S3 Supplementary Tables

Table S1 The values of Ni 2p for different samples

**Catalysts**	**Ni^0 $2p_{3/2}$ (eV)**	**Ni^0 $2p_{1/2}$ (eV)**
Ni/MoO_2@CN	853.0	870.3
Ni/MoO_2	852.7	870.0
Ni@CN	852.5	869.8

Table S2 The values of Mo 3d for different samples

**Catalysts**	**Mo^{4+} $3d_{5/2}$ (eV)**	**Mo^{4+} $3d_{3/2}$ (eV)**
Ni/MoO_2@CN	229.4	232.5
Ni/MoO_2	229.7	232.8
MoO_2@CN	229.9	233.0

Table S3 Comparisons of HER activity of Ni/MoO_2@CN with other reported non-noble-metal catalysts

**Catalysts**	**$\eta_{-1,000}$ (mV)**	**Refs.**
**Ni/MoO_2@CN**	**267**	**This work**
NiP_2-FeP_2	327	[S16]
Ni_2P-Fe_2P/NF	333	[S17]
$Ni_{2(1-x)}Mo_{2x}P$	294	[S18]
C-$Ni_{1-x}O$/3DPNi	245	[S19]
Co-Ni_3S_2/NF	750	[S20]
FeP/Ni_2P	~275	[S21]
MoS_2/Mo_2C	220	[S22]
$F_{0.25}C_1$CH/NF	256	[S23]
Sn-Ni_3S_2/NF	570	[S24]
Ni_2P/NF	306	[S25]

Table S4 Comparisons of TOF values of Ni/MoO_2@CN, Ni/MoO_2, MoO_2@CN and Ni@CN for HER

**Catalysts**	**TOF (s^{-1}@100 mV)**
Ni/MoO_2@CN	1.45
Ni/MoO_2	0.53
MoO_2@CN	0.38
Ni@CN	0.19

Table S5 TOF values of Ni/MoO_2@CN obtained at different overpotentials for HER

**Overpotentials (mV)**	**TOF (s^{-1})**
50	0.51
100	1.45
150	3.18

Table S6 Comparisons of TOF values of Ni/MoO_2@CN for HER with other reported non-noble-metal catalysts

**Catalysts**	**TOF (s^{-1}@ mV)**	**Refs.**
**Ni/MoO_2@CN**	**1.45@100**	**This work**
Mo_2N-Mo_2C/HGr	0.086@100	[S26]
Ni_2P	0.012@100	[S27]
Co@N-CNT/NF	0.75@100	[S8]
MoS_3-CV films	0.3@340	[S6]
Ni_3N-VN/NF	1.5@100	[S7]
P-Fe_3N@NC NSs/IF	~0.7@100	[S28]
N-NiVFeP/NFF	~0.04@180	[S29]
NPC-sheet@NF	0.89@200	[S9]
Ni_9S_8@MoS_2	0.5@84	[S10]
Mn-MoS_2/rGO	0.05@110	[S30]
MoS_2/$NiCo_2S_4$	0.5@89	[S31]

Table S7 Comparisons of OER activity of Ni/MoO_2@CN with other reported non-noble-metal catalysts

Catalysts	$\eta_{1,000}$ (mV)	Refs.
**Ni/MoO_2@CN**	**420**	**This work**
KT-Ni(0)@Ni(II)-TPA	~370	[S32]
Ni_2P-Fe_2P/NF	337	[S17]
Ni-Fe-OH@Ni_3S_2/NF	~720	[S33]
Fe-CoP/NF	428	[S34]
C-$Ni_{1-x}O$/3DPNi	425	[S19]
(Ni-Fe)S_x/NiFe$(OH)_y$	510	[S35]

Co-Ni_3S_2/NFs	850	[S20]
Sn-Ni_3S_2/NF	570	[S24]
Co_1Mn_1CH/NF	462	[S36]
CuS-Ni_3S_2/CuNi/NF	510	[S37]

Table S8 Comparisons of TOF values of Ni/MoO_2@CN, Ni/MoO_2 and Ni@CN for OER

**Catalysts**	**TOF (s^{-1}@ 300 mV)**
Ni/MoO_2@CN	1.23
Ni/MoO_2	0.28
Ni@CN	0.14

Table S9 TOF values of Ni/MoO_2@CN obtained at different overpotentials for OER

**Overpotentials (mV)**	**TOF (s^{-1})**
250	0.21
300	1.23
350	4.9

Table S10 Comparison of TOF values of Ni/MoO_2@CN for OER with other reported non-noble-metal catalysts

**Catalysts**	**TOF (s^{-1}@ mV)**	**Reference**
**Ni/MoO_2@CN**	**1.23@300**	**This work**
FCN-MOF/NF	0.865@250	[S38]
NiS_2/$NiSe_2$	~0.065@300	[S12]
NiMoN@NiFeN	0.09@300	[S14]
$Ni(OH)_2$-TCNQ/CF	0.24@400	[S13]
Ni_3N-VN/NF	1.52@300	[S7]
Ni_3N-COF	0.52@300	[S39]
FeCoW	0.46@300	[S40]
Co-Se NSs	~1.14@300	[S41]
Zn-Co-LDH	0.88@710	[S42]
HFC Co_3O_4-250	~0.018@400	[S11]
NiFe-NS	0.05@300	[S43]

S4 Supplementary References

[S1] J.P. Perdew, K. Burke, M. Ernzerhof, Generalized gradient approximation made simple, Phys. Rev. Lett. **77**, 3865-3868 (1996). https://doi.org/10.1103/PhysRevLett.77.3865

[S2] B. Hammer, L.B. Hansen, J.K. Nørskov, Improved adsorption energetics within density-functional theory using revised Perdew-Burke-Ernzerhof functionals. Phy. Rev. B **59**, 7413-7421 (1999). https://doi.org/10.1103/PhysRevB.59.7413

[S3] P.E. Blöchl, Projector augmented-wave method. Phy. Rev. B **50**, 17953-17979 (1994). https://doi.org/10.1103/PhysRevB.50.17953

[S4] G. Kresse, D. Joubert, From ultrasoft pseudopotentials to the projector augmented-wave method. Phys. Rev. B **59**, 1758-1775 (1999). https://doi.org/10.1103/PhysRevB.59.1758

[S5] H.J. Monkhorst, J.D. Pack, Special points for Brillouin-zone integrations. Phy. Rev. B **13**, 5188-5192 (1976). https://doi.org/10.1103/PhysRevB.16.1746

[S6] D. Merki, S. Fierro, H. Vrubel, X.L. Hu, Amorphous molybdenum sulfide films as catalysts for electrochemical hydrogen production in water. Chem. Sci. **2**, 1262-1267 (2011). https://doi.org/10.1039/c1sc00117e

[S7] H.J. Yan, Y. Xie, A.P. Wu, Z.C. Cai, L. Wang et al., Anion-modulated HER and OER activities of 3D Ni-V-based interstitial compound heterojunctions for high-efficiency and stable overall water splitting. Adv. Mater. **31**, 1901174 (2019). https://doi.org/10.1002/adma.201901174

[S8] L.J. Yang, H. Li, Y. Yu, Y. Wu, L. Zhang, Assembled 3D MOF on 2D nanosheets for self-boosting catalytic synthesis of N-doped carbon nanotube encapsulated metallic Co electrocatalysts for overall water splitting. Appl. Catal. B **271**, 118939 (2020). https://doi.org/10.1016/j.apcatb.2020.118939

[S9] H.Q. Qu, Y.R. Ma, Z.L. Gou, B. Li, Y.R. Liu et al., Ni_2P/C nanosheets derived from oriented growth Ni-MOF on nickel foam for enhanced electrocatalytic hydrogen evolution. J. Colloid Interface Sci. **572**, 83-90 (2020). https://doi.org/10.1016/j.jcis.2020.03.068

[S10] X.B. Xu, W. Zhong, L. Zhang, G.X. Liu, Y.W. Du, Synergistic effect of MoS_2 and Ni_9S_8 nanosheets as an efficient electrocatalyst for hydrogen evolution reaction. J. Colloid Interface Sci. **556**, 24-32 (2019). https://doi.org/10.1016/j.jcis.2019.08.041

[S11] X.M. Zhou, X.T. Shen, Z.M. Xia, Z.Y. Zhang, J. Li et al., Hollow fluffy Co_3O_4 cages as efficient electroactive materials for supercapacitors and oxygen evolution reaction. ACS Appl. Mater. Interfaces **7**, 20322-20331 (2015). https://doi.org/10.1021/acsami.5b05989

[S12] Y. Yang, Y.K. Kang, H.H. Zhao, X.P. Dai, M.L. Cui et al., An interfacial electron transfer on tetrahedral NiS_2/$NiSe_2$ heterocages with dual-phase synergy for efficiently triggering the oxygen evolution reaction. Small **16**, 1905083 (2020). https://doi.org/10.1002/smll.201905083

[S13] X.X. Guo, R.M. Kong, X.P. Zhang, H.T. Du, F.L. Qu, $Ni(OH)_2$ nanoparticles embedded in conductive microrod array: An efficient and durable electrocatalyst for alkaline oxygen evolution reaction. ACS Catal. **8**, 651-655 (2017). https://doi.org/10.1021/acscatal.7b03406

[S14] L. Yu, Q. Zhu, S.W. Song, B. McElhenny, D.Z. Wang et al., Non-noble metal-nitride based electrocatalysts for high-performance alkaline seawater electrolysis. Nat.

Commun. **10**, 5106 (2019). https://doi.org/10.1038/s41467-019-13092-7

[S15] F. Zhang, R.J. Ji, Y.H. Liu, Y. Pan, B.P. Cai et al., A novel nickel-based honeycomb electrode with microtapered holes and abundant multivacancies for highly efficient overall water splitting. Appl. Catal., B **276**, 119141 (2020). https://doi.org/10.1016/j.apcatb.2020.119141

[S16] A. Kumar, V.Q. Bui, J.Y. Lee, A.R. Jadhav, Y. Hwang et al., Modulating interfacial charge density of NiP_2-FeP_2 via coupling with metallic Cu for accelerating alkaline hydrogen evolution. ACS Energy Lett. **6**, 354-363 (2021). https://doi.org/10.1021/acsenergylett.0c02498

[S17] L.B. Wu, L. Yu, F.H. Zhang, B. McElhenny, D. Luo et al., Heterogeneous bimetallic phosphide Ni_2P-Fe_2P as an efficient bifunctional catalyst for water/seawater splitting. Adv. Funct. Mater. **31**, 2006484 (2020). https://doi.org/10.1002/adfm.202006484

[S18] L. Yu, I.K. Mishra, Y.L. Xie, H.Q. Zhou, J.Y. Sun et al., Ternary $Ni_{2(1-x)}Mo_{2x}P$ nanowire arrays toward efficient and stable hydrogen evolution electrocatalysis under large-current-density. Nano Energy **53**, 492-500 (2018). https://doi.org/10.1016/j.nanoen.2018.08.025

[S19] T.Y. Kou, S.W. Wang, R.P. Shi, T. Zhang, S. Chiovoloni et al., Periodic porous 3D electrodes mitigate gas bubble traffic during alkaline water electrolysis at high current densities. Adv. Energy Mater. **10**, 2002955 (2020). https://doi.org/10.1002/aenm.202002955

[S20] J. Jian, L. Yuan, H. Li, H.H. Liu, X.H. Zhang et al., Hydrothermal synthesized Co-Ni_3S_2 ultrathin nanosheets for efficient and enhanced overall water splitting. Chem. Res. Chin. Univ. **35**, 179-185 (2019). https://doi.org/10.1007/s40242-019-8344-x

[S21] F. Yu, H.Q. Zhou, Y.F. Huang, J.Y. Sun, F. Qin et al., High-performance bifunctional porous non-noble metal phosphide catalyst for overall water splitting. Nat. Commun. **9**, 2551 (2018). https://doi.org/10.1038/s41467-018-04746-z

[S22] Y.T. Luo, L. Tang, U. Khan, Q.M. Yu, H.M. Cheng et al., Morphology and surface chemistry engineering toward pH-universal catalysts for hydrogen evolution at high current density. Nat. Commun. **10**, 269 (2019). https://doi.org/10.1038/s41467-018-07792-9

[S23] L. Hui, Y.R. Xue, D.Z. Jia, H.D. Yu, C. Zhang et al., Multifunctional single-crystallized carbonate hydroxides as highly efficient electrocatalyst for full water splitting. Adv. Energy Mater. **8**, 1800175 (2018). https://doi.org/10.1002/aenm.201800175

[S24] J. Jian, L. Yuan, H. Qi, X.J. Sun, L. Zhang et al., Sn-Ni_3S_2 ultrathin nanosheets as efficient bifunctional water-splitting catalysts with a large current density and low overpotential. ACS Appl. Mater. Interfaces **10**, 40568-40576 (2018). https://doi.org/10.1021/acsami.8b14603

[S25] X.X. Yu, Z.Y. Yu, X.L. Zhang, Y.R. Zheng, Y. Duan et al., "Superaerophobic" nickel

phosphide nanoarray catalyst for efficient hydrogen evolution at ultrahigh current densities. J. Am. Chem. Soc. **141**, 7537-7543 (2019). https://doi.org/10.1021/jacs.9b02527

[S26] H.J. Yan, Y. Xie, Y.Q. Jiao, A.P. Wu, C.G. Tian et al., Holey reduced graphene oxide coupled with an Mo_2N-Mo_2C heterojunction for efficient hydrogen evolution. Adv. Mater. **30**, 1704156 (2018). https://doi.org/10.1002/adma.201704156

[S27] E.J. Popczun, J.R. McKone, C.G. Read, A.J. Biacchi, A.M. Wiltrout et al., Nanostructured nickel phosphide as an electrocatalyst for the hydrogen evolution reaction. J. Am. Chem. Soc. **135**, 9267-9270 (2013). https://doi.org/10.1021/ja403440e

[S28] G.X. Li, J.Y. Yu, W.Q. Yu, L.J. Yang, X.L. Zhang et al., Phosphorus-doped iron nitride nanoparticles encapsulated by nitrogen-doped carbon nanosheets on iron foam in situ derived from saccharomycetes cerevisiae for electrocatalytic overall water splitting. Small **16**, 2001980 (2020). https://doi.org/10.1002/smll.202001980

[S29] H.F. Fan, W. Chen, G.L. Chen, J. Huang, C.S. Song et al.,Plasma-heteroatom-doped Ni-V-Fe trimetallic phospho-nitride as high-performance bifunctional electrocatalyst. Appl. Catal. B **268**, 118440 (2020). https://doi.org/10.1016/j.apcatb.2019.118440

[S30] L.Q. Wu, X.B. Xu, Y.Q. Zhao, K.Y. Zhang, Y. Sun et al., Mn doped MoS_2/reduced graphene oxide hybrid for enhanced hydrogen evolution. Appl. Surf. Sci. **425**, 470-477 (2017). https://doi.org/10.1016/j.apsusc.2017.06.223

[S31] X.B. Xu, W. Zhong, S.L. Yan, L. Zhang, G.X. Liu et al., Advanced catalysts for hydrogen evolution reaction based on MoS_2/$NiCo_2S_4$ heterostructures in alkaline media. Int. J. Hydrogen Energy **45**, 1759-1768 (2020). https://doi.org/10.1016/j.ijhydene.2019.11.045

[S32] Q. Hu, Z.Y. Wang, X.W. Huang, Y.J. Qin, H.P. Yang et al., Integrating well-controlled core-shell structures into “superaerophobic” electrodes for water oxidation at large current densities. Appl. Catal., B **286**, 119920 (2021). https://doi.org/10.1016/j.apcatb.2021.119920

[S33] X. Zou, Y.P. Liu, G.D. Li, Y.Y. Wu, D.P. Liu et al., Ultrafast formation of amorphous bimetallic hydroxide films on 3D conductive sulfide nanoarrays for large-current-density oxygen evolution electrocatalysis. Adv. Mater. **29**, 1700404 (2017). https://doi.org/10.1002/adma.201700404

[S34] L.M. Cao, Y.W. Hu, S.F. Tang, A. Iljin, J.W. Wang et al., Fe-CoP electrocatalyst derived from a bimetallic prussian blue analogue for large-current-density oxygen evolution and overall water splitting. Adv. Sci. **5**, 1800949 (2018). https://doi.org/10.1002/advs.201800949

[S35] Q.J. Che, Q. Li, Y. Tan, X.H. Chen, X. Xu et al., One-step controllable synthesis of amorphous (Ni-Fe)S_x/NiFe(OH) hollow microtube/sphere films as superior bifunctional electrocatalysts for quasi-industrial water splitting at large-current-density. Appl. Catal., B **246**, 337-348 (2019). https://doi.org/10.1016/j.apcatb.2019.01.082

[S36] T. Tang, W.J. Jiang, S. Niu, N. Liu, H. Luo et al., Electronic and morphological dual modulation of cobalt carbonate hydroxides by Mn doping toward highly efficient and stable bifunctional electrocatalysts for overall water splitting. J. Am. Chem. Soc. **139**, 8320-8328 (2017). https://doi.org/10.1021/jacs.7b03507

[S37] N. Zhang, Y. Gao, Y.H. Mei, J. Liu, W.Y. Song et al., CuS-Ni_3S_2 grown in situ from three-dimensional porous bimetallic foam for efficient oxygen evolution. Inorg. Chem. Front. **6**, 293-302 (2019). https://doi.org/10.1039/c8qi01148f

[S38] D. Senthil Raja, C.L. Huang, Y.A. Chen, Y.M. Choi, S.Y. Lu, Composition-balanced trimetallic MOFs as ultra-efficient electrocatalysts for oxygen evolution reaction at high current densities. Appl. Catal., B **279**, 119375 (2020). https://doi.org/10.1016/j.apcatb.2020.119375

[S39] S. Nandi, S.K. Singh, D. Mullangi, R. Illathvalappil, L. George et al., Low band gap benzimidazole COF supported Ni_3N as highly active OER catalyst. Adv. Energy Mater. **6**, 1601189 (2016). https://doi.org/10.1002/aenm.201601189

[S40] B. Zhang, X.L. Zheng, O. Voznyy, R. Comin, M. Bajdich et al., Homogeneously dispersed multimetal oxygen-evolving catalysts. Science **352**, 333-337 (2016). https://doi.org/10.1126/science.aaf1525

[S41] Y.Z. Xue, Z.Y. Ren, Y. Xie, S.C. Du, J. Wu et al., $CoSe_x$ nanocrystalline-dotted CoCo layered double hydroxide nanosheets: A synergetic engineering process for enhanced electrocatalytic water oxidation. Nanoscale **9**, 16256-16263 (2017). https://doi.org/10.1039/c7nr05867e

[S42] X.X. Zou, A. Goswami, T. Asefa, Efficient noble metal-free(electro)catalysis of water and alcohol oxidations by zinc-cobalt layered double hydroxide. J. Am. Chem. Soc. **135**, 17242-17245 (2013). https://doi.org/10.1021/ja407174u

[S43] F. Song, X.L. Hu, Exfoliation of layered double hydroxides for enhanced oxygen evolution catalysis. Nat. Commun. **5**, 4477 (2014). https://doi.org/10.1038/ncomms5477
